# Stabilizing Na_0.7_MnO_2_ Cathodes in PEO-Based Sodium Metal Batteries via Composite Interlayer Design

**DOI:** 10.1021/acsomega.6c06046

**Published:** 2026-08-04

**Authors:** Inbar Anconina, Thomas Leirikh, Diana Golodnitsky

**Affiliations:** School of Chemistry, Faculty of Exact Sciences, 26745 Tel Aviv University, Tel Aviv 6997801, Israel

## Abstract

Poly­(ethylene oxide) (PEO)-based solid polymer electrolytes are attractive for sodium metal batteries but suffer from interfacial instability when coupled with layered oxide cathodes. Here, sodium β″-alumina (SBA) was tested as an ion-conducting filler in a PEO-based composite solid polymer electrolyte (CSPE) and as a major component of a composite cathode interlayer deposited via electrophoretic deposition using a polymeric ionic liquid (PIL). Although the solid polymer electrolyte without ceramic filler and the CSPE exhibited comparable ionic conductivity at 60 °C, the CSPE displayed lower activation energy and improved cell durability, underscoring the importance of ceramic reinforcement beyond bulk transport considerations. Within this stabilized CSPE platform, the SBA–PIL interlayer effectively regulated the cathode-electrolyte interface of layered Na_0.7_MnO_2_. As a result, polarization growth was suppressed, Coulombic efficiency increased (99.1% vs 97.9%), and capacity retention was significantly improved (76% vs 46% after 50 cycles) compared to pristine cathodes. Impedance analysis further revealed moderated charge-transfer resistance evolution and more stable interfacial kinetics. These findings demonstrate that combining ceramic-reinforced polymer electrolytes with targeted cathode interfacial engineering is an effective strategy to mitigate degradation in PEO-based sodium metal solid polymer batteries.

## Graphical abstract

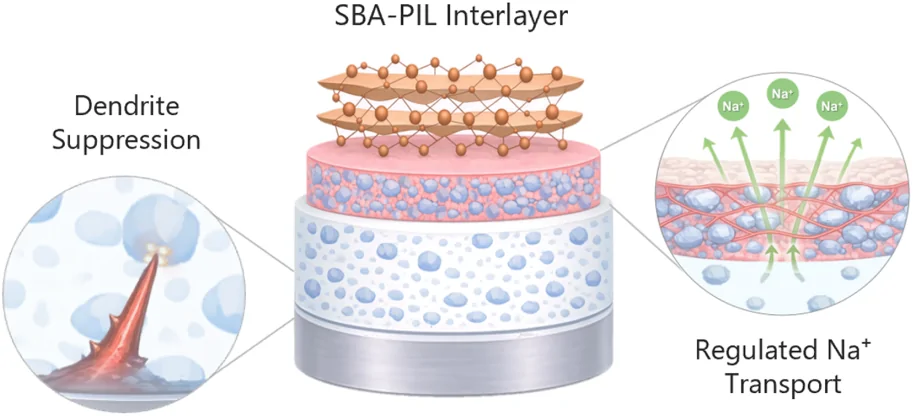

## 1 Introduction

Solid-state alkali-ion batteries employing solid polymer electrolytes (SPEs) have garnered growing interest due to their intrinsic safety, mechanical flexibility, and compatibility with scalable manufacturing.^1^ Among SPE candidates, poly­(ethylene oxide) (PEO)-based electrolytes are particularly attractive, enabling thin and mechanically robust membranes with good salt solubility and intimate electrode contact at moderately elevated temperatures.^2,3^ Extending PEO-based polymer electrolytes to sodium batteries leverages the earth abundance and low cost of sodium while mitigating the safety hazards and sodium–metal interfacial instability associated with flammable carbonate-based liquid electrolytes.^4–7^ As a result, PEO-based SPEs in sodium metal batteries represent a promising route toward safe and sustainable large-scale energy storage.^8–10^

Despite these advantages, the practical implementation of PEO-based SPEs in batteries remains challenging due to a high degree of crystallinity at ambient temperatures, which restricts segmental motion, ionic conductivity, and Na^+^ transference number.^1,2,11^ Moreover, PEO-based electrolytes exhibit limited intrinsic oxidative stability and their effective stability in cells is strongly influenced by cathode surface reactivity, as cathode-driven interfacial reactions can catalyze electrolyte oxidation at low potentials.^12–15^ This process leads to continuous cathode-electrolyte interphase (CEI) degradation and electrolyte decomposition, ultimately driving progressive capacity fade and long-term performance degradation.^16–18^

Degradation processes initiated at the cathode/electrolyte interface can subsequently propagate throughout the cell via coupled mechanisms and interfere with solid electrolyte interphase (SEI) formation at the sodium metal anode.^18–21^ Together with the low Na^+^ transference number of PEO-based electrolytes, these effects promote strong concentration gradients during cycling, resulting in nonuniform Na^+^ flux, unstable SEI growth, and localized sodium deposition that promotes dendrite nucleation and growth.^22,23^ The coupling between cathode-side parasitic reactions and anode-side dendritic failure mechanisms thus establishes a mutually reinforcing degradation cascade that ultimately deteriorates cell lifetime.

To mitigate these challenges, various strategies have been proposed to improve the ionic conductivity and electrochemical stability of PEO-based SPEs.^24^ They include synthesis or blending with amorphous copolymers featuring high-voltage stability,^25,26^ and the use of various additives to suppress crystallization and oxidative and interfacial degradation.^27,28^ Among these approaches, the incorporation of ceramic fillers has emerged as particularly promising.^29–33^ Ion-conducting ceramic fillers that can simultaneously enhance ionic conductivity, improve mechanical integrity, and moderate interfacial reactivity are especially noteworthy.^34–36^ Nonetheless, these strategies often provide insufficient protection against cathode-induced interfacial degradation, particularly when chemically reactive cathode materials are employed.^14,37^ Cathode surface coatings,^38–41^ or cathode-electrolyte interlayers,^42,43^ that decouple the reactive oxide surface from the polymer electrolyte are considered as promising approaches to tackle the stability challenge.

Many prior sodium-based studies have relied on structurally robust, but comparatively expensive cathode materials like NASICON-type Na_3_V_2_(PO_4_)_3_,^44–46^ whose rigid three-dimensional frameworks and low surface reactivity afford enhanced interfacial stability with polymer electrolytes, making them valuable model platforms.^34,35,47^ Layered transition-metal oxides, such as Na_0.7_MnO_2_ (NMO), offer lower cost and higher energy density, but exhibit pronounced structural instability and surface reactivity associated with lattice oxygen activity and surface reconstruction under high-voltage charging conditions.^48–51^ These characteristics render layered oxides particularly sensitive to interfacial polarization and electrolyte decomposition even at relatively low operating voltages.

Herein, we employ sodium β″-alumina (SBA) both as an ion-conducting filler in a PEO-based composite solid polymer electrolyte (CSPE) and as a functional component of a cathode interlayer (Figure 1). In sodium systems, SBA is a well-established fast Na^+^ conductor and has been widely reported to provide continuous ion-transport pathways and enhanced interfacial robustness when incorporated into polymer matrices or composite architectures.^52–54^ The interlayer is deposited via electrophoretic deposition (EPD),^55,56^ using a polymerized ionic liquid (PIL) that serves simultaneously as a charging agent and a binder, an approach previously demonstrated by us in lithium-ion systems employing liquid electrolytes.^57,58^ Here, this concept is extended to sodium metal solid polymer batteries, enabling the formation of a conformal SBA–PIL-based interlayer on layered NMO cathodes. By combining bulk electrolyte modification with targeted cathode interface engineering, this work aims to demonstrate the effectiveness of interlayer-assisted interfacial regulation, and to elucidate the role of ceramic fillers and interlayers in mitigating cathode-induced interfacial degradation in PEO-based sodium metal solid batteries.

**Figure 1:**
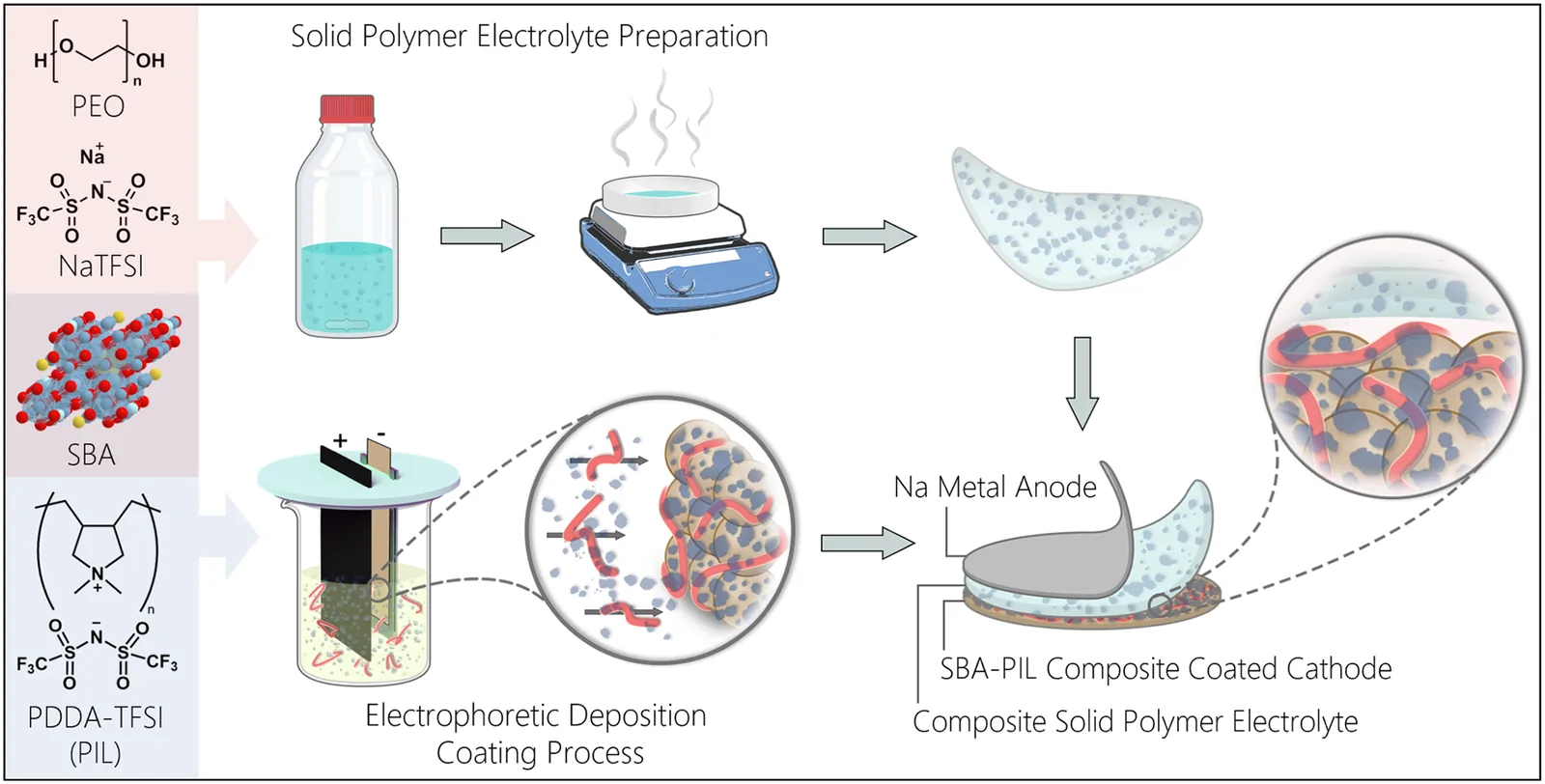
Schematic overview of the composite solid polymer electrolyte (CSPE) and cathode interlayer design for sodium metal solid polymer cells. Sodium β″-alumina (SBA; crystal structure adapted from The Materials Project^96,97^ ) is incorporated as an ion-conducting filler in a PEO-NaTFSI solid polymer electrolyte and deposited as an SBA–PIL composite interlayer on the Na_0.7_MnO_2_ cathode via electrophoretic deposition.

## 2 Results and Discussion

### 2.1 Structural and Morphological Characterization of the CSPE

NaTFSI-PEO polymer electrolytes with Na:EO molar ratio of 1:18 used for cell assembly were free-standing films that retained mechanical integrity when handled with tweezers, as shown in the inset photographs of Figure 2a. Free-of-filler SPE (0 wt % SBA) and CSPEs with 5 and 10 wt % SBA were prepared by casting and characterized for comparison. The 5 wt % SBA NaTFSI-PEO composition (CSPE-5) was selected for detailed analysis based on its favorable mechanical robustness and electrochemical performance.

**Figure 2:**
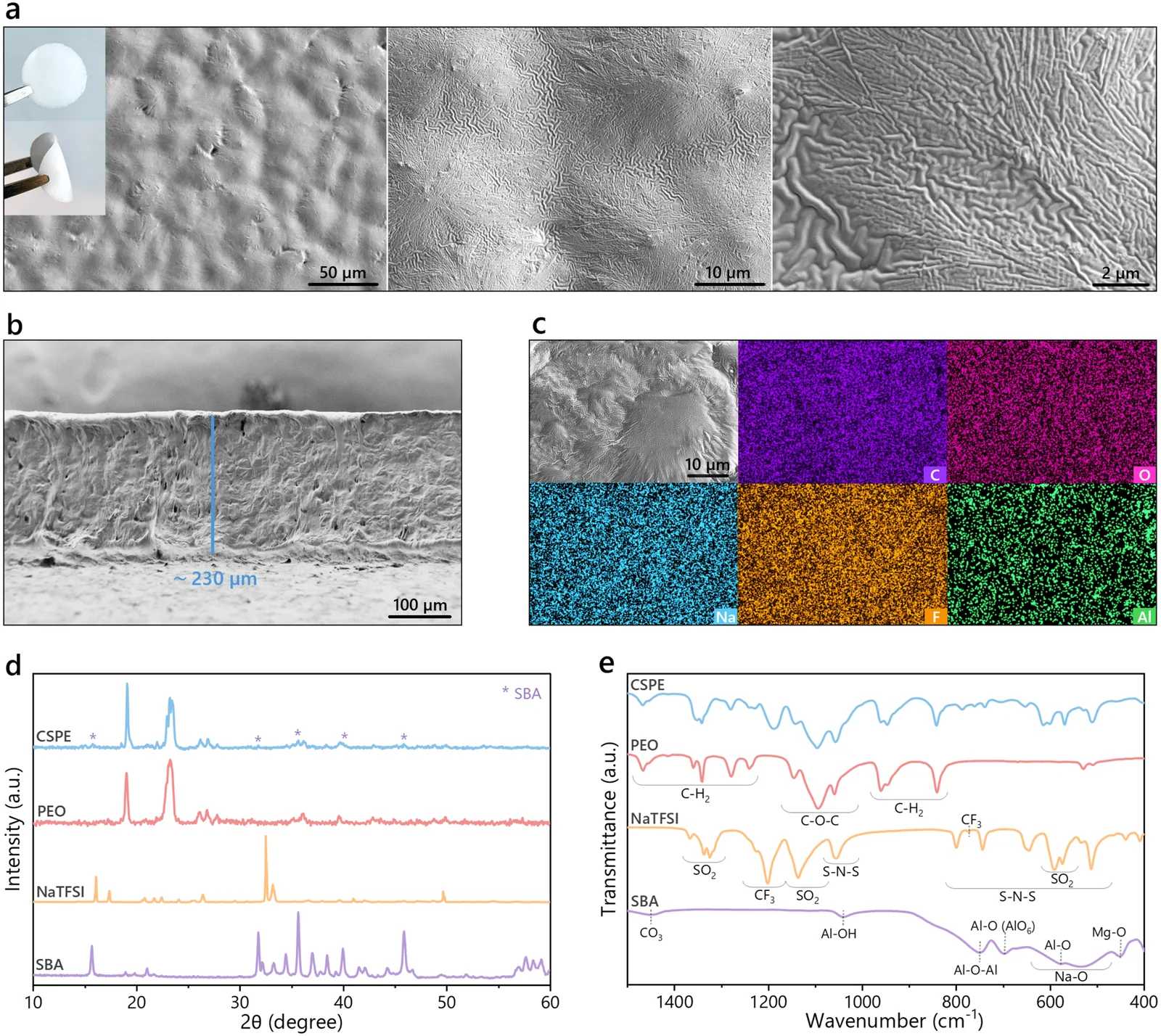
Morphological, structural, and chemical characterization of the CSPE-5. (a) Surface HRSEM images at increasing magnification (inset photographs show the free-standing film). (b) Cross-sectional HRSEM image of the cast film showing a dense interior with a thickness of ∼230 μm. (c) SEM image and corresponding EDS elemental maps. (d) XRD patterns of SBA, NaTFSI, PEO, and CSPE. (e) FTIR spectra of the individual components and CSPE.

High-resolution scanning electron microscopy (HRSEM) images revealed that the filler-free SPE exhibited large, well-defined spherulites (Figure S1). Incorporation of 5 wt % SBA significantly refined the microstructure, yielding smaller and more interconnected domains, while 10 wt % SBA maintained the reduced spherulite size. The CSPE-5 composite electrolyte exhibited a multispherulitic surface morphology composed of banded, dome-shaped domains (∼10–30 μm) separated by narrow boundaries (Figure 2a). This morphology is characteristic of semicrystalline polymers, appearing during solvent evaporation.^59,60^ The relatively small spherulite size is attributed to complexation of sodium by ethylene oxide units, which suppresses crystal growth and lowers effective PEO crystallinity. Heterogeneous, high-density nucleation induced by SBA particles is likely to contribute to the observed morphology, as well.^29,61,62^ At higher magnification, the spherulites consisted of quasi-radially arranged lamellar bundles, with denser packing at the spherulite centers and more open, deflected structures at interspherulitic boundaries. Such microstructural refinement suggests a reduced crystalline fraction and enhanced interfacial conduction pathways that favor ion transport.^63^ Cross-sectional imaging of this composite electrolyte revealed a dense interior without discernible voids and an average film thickness of ∼230 μm (Figure 2b).

Energy-dispersive X-ray spectroscopy (EDS) elemental maps indicated a spatially uniform distribution of the constituents at the micrometer scale in the CSPE-5 (Figure 2c). Fluorine derived from NaTFSI and sodium were homogeneously dispersed throughout the matrix, with sodium associated primarily with the salt and, to a lesser extent, with the SBA ceramic. Aluminum associated with the SBA filler was uniformly distributed with no visible agglomeration. No phase-segregated regions were detected within the spatial resolution of the maps.

The X-ray diffraction (XRD) patterns of the individual components and the CSPE-5 sample are shown in Figure 2d. Pristine PEO exhibited two intense diffraction peaks at approximately 19.0° and 23.2°, assigned to the (120) and overlapping (112)/(032) reflections, respectively (PDF 47-2095), arising from the ordered arrangement of helical PEO chains in a monoclinic unit cell, along with weaker reflections at higher angles. The crystalline phase coexists with an amorphous one resulting to PEO designation as a semicrystalline polymer matrix. NaTFSI displayed multiple sharp reflections confirming its highly crystalline nature. SBA exhibited a distinct diffraction pattern composed of numerous sharp reflections, notably at ∼15.7°, 31.8°, 35.6°, 40.0°, and 45.8°. Phase identification indicated that the SBA pattern corresponded predominantly to Mg-doped sodium β″-alumina, in which Mg partially substituted for Al in the framework (Na_1.65_Mg_0.65_Al_10.35_O_17_, PDF 73-9550). Weaker overlapping reflections are attributed to β′-alumina (NaAl_11_O_17_, PDF 21-1096) (Figure S2). The β″ phase, with its layered hexagonal structure containing fast Na^+^ conduction planes, is well suited as a ceramic ion conductor.^52^

In the CSPE-5 diffraction pattern, the two main PEO crystalline reflections were retained, however, their relative intensities were inverted compared to neat PEO, with the 19.1° peak becoming more intense than the 23.2° peak. This inversion suggests modification of crystallite orientation and packing, likely induced by Na^+^ coordination to PEO ether oxygens and interactions with the SBA filler.^64,65^ The sharp NaTFSI reflections disappeared, indicating that the neat salt entities were not present in the composite, on the contrary, the salt dissociated and that the amorphous complexes were formed between sodium cation and oxygens of the ethylene oxide group. Weak but discernible SBA reflections persisted, consistent with its lower loading and attenuation by the polymer-salt matrix. Overall, the partial retention yet reduced prominence of crystalline features, together with the inverted PEO peak ratio, indicates polymer-phase reorganization that is favorable for Na^+^ transport through enhanced segmental mobility.

Fourier-transform infrared spectroscopy (FTIR) was employed to probe the chemical structure and interactions within the composite electrolyte (Figure 2e). Neat PEO exhibited characteristic CH_2_ bending modes at 1467, 1360, and 1342 cm^–1^, and twisting modes at 1278 and 1240 cm^–1^, with a weaker shoulder at 1234 cm^–1^, reflecting variations in chain conformation and local symmetry within the semicrystalline polymer. The ether backbone was clearly identified by the C–O–C stretching vibrations at 1146, 1095, and 1059 cm^–1^, which are characteristic of the PEO chain structure.^66–68^

Upon incorporation of NaTFSI and SBA, the PEO backbone features were retained, indicating structural integrity of the polymer matrix, while additional bands and subtle shifts emerged, primarily reflecting polymer-salt interactions. The most significant changes occur in the C–O stretching region. As the cation interacts with the oxygen atoms, the electron density of the C–O–C bond is altered, leading to a shift in the asymmetric stretching mode around 1100–1070 cm^–1^ due to the ion-dipole interaction. Shifts of 1466 and 1452 cm^–1^ bands were observed as a result of changes in the local environment of the polymer chain, specifically the gauche–trans conformational changes of the ethylene oxide unit upon Na^+^ binding. A new feature near ∼1350 cm^–1^ was assigned to shifted C–H bending modes overlapping with SO_2_ vibrations, perturbed by Na^+^-ether coordination. Additional bands associated with TFSI^–^ appeared, including CF_3_ symmetric stretching at ∼1190 cm^–1^ and asymmetric S–N–S stretching at 1057 cm^–1^. In the 650–500 cm^–1^ region, S–N–S bending modes were observed with slight displacement. Shifts relative to neat NaTFSI suggest modification of the anion coordination environment due to Na^+^-ether interactions and increased local polarity. In the far-infrared region, the formation of the complex typically creates a new IR signature, often described as a “cage” vibration where the Na^+^ cation is coordinated to several ether oxygens like in sodium crown-ether and lithium-PEO complexes.^69,70^ However, a strong overlapping of the IR bands of composite electrolyte components does not enable precise correlation of the bands. The SBA contribution was identified by the Al–O stretching band near 705 cm^–1^. Features associated with Na–O and Mg–O vibrations (a broad band centered around 534 cm^–1^) were weak or partially overlapped due to the low filler content.^71–74^ Overall, FTIR confirms preservation of the PEO backbone together with modified Na^+^/TFSI^–^ coordination within the polymer-salt-ceramic network.

### 2.2 Electrochemical Properties of Na-Ion Conducting CSPE

Electrochemical impedance spectroscopy (EIS) measurements were performed over a range of temperatures using symmetric Na/CSPE-5/Na cells to evaluate bulk and interfacial transport processes. Representative EIS spectra and the corresponding DRT analysis are shown for a cell with behavior close to the average trend, while the extracted conductivity and SEI resistivity values are summarized statistically from three independent cells. A representative Nyquist plot at 40 °C and the corresponding equivalent circuit are shown in Figure 3a. The equivalent circuit comprised a bulk resistance (*R_B_*), followed by serial *R*–*Q* (constant phase) elements describing grain-boundary and interfacial processes, and a finite-length Warburg element representing diffusion contributions in the CSPE.^75–77^ The extracted fitting parameters as a function of temperature are summarized in Table S1.

**Figure 3:**
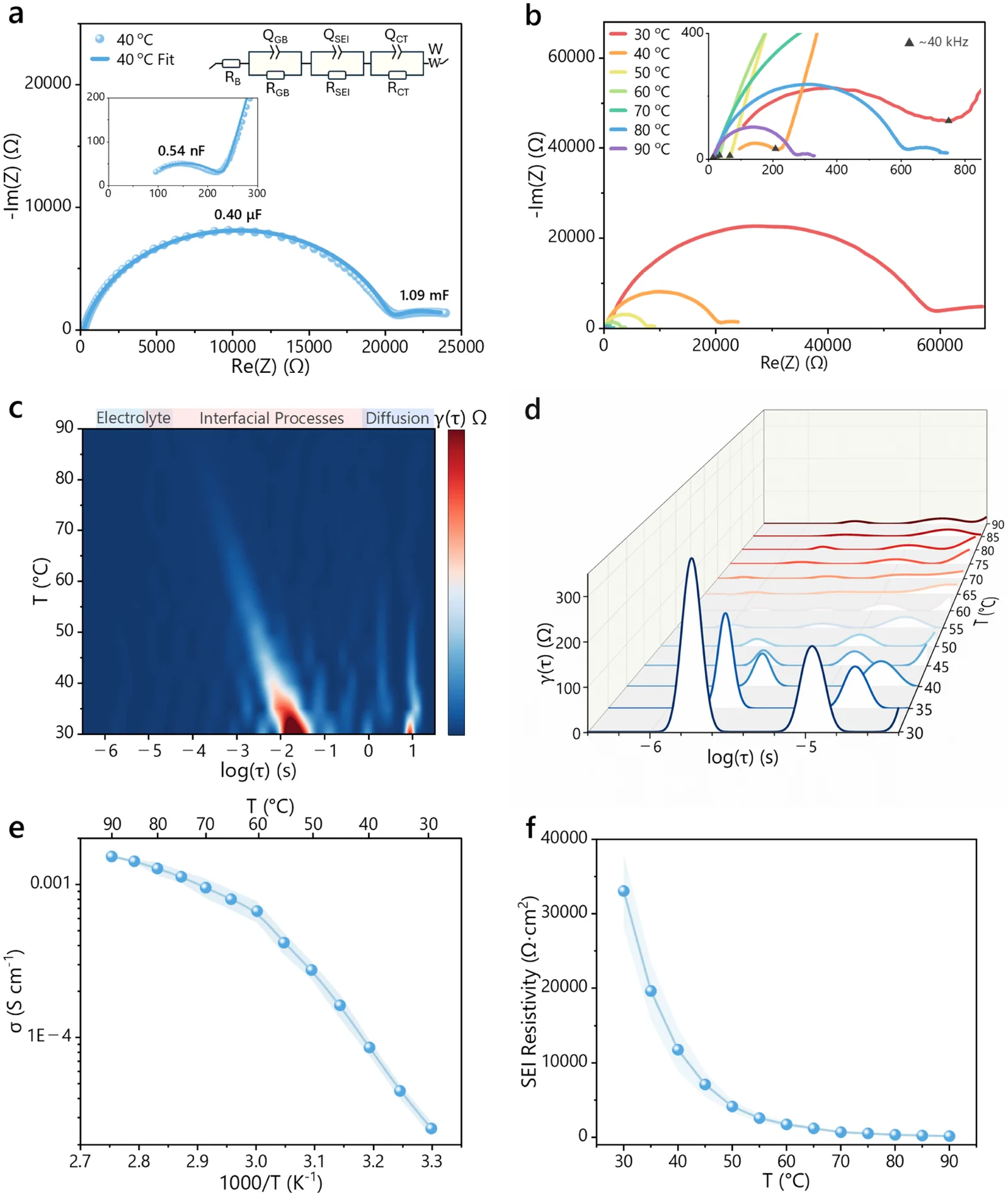
Electrochemical characterization of the CSPE-5 in symmetric Na/CSPE/Na cells. (a) Representative Nyquist plot at 40 °C with equivalent circuit used for fitting (inset). (b) Temperature-dependent Nyquist spectra. (c) Temperature-dependent DRT contour map showing the evolution of electrolyte and electrode contributions with increasing temperature. (d) Stacked DRT spectra highlighting the high-frequency region and the evolution of bulk and grain-boundary processes with temperature. (e) Ionic conductivity derived from *R_B_* + *R_GB_*. (f) Temperature dependence of SEI resistivity extracted from impedance fitting. Data in (e, f) are presented as mean ± standard deviation from three independent cells.

The high-frequency intercept of the Nyquist plot with the real axial corresponds to *R_B_*, representing bulk electrolyte resistance. A high-frequency semicircle with *f_max_* in the ∼0.9–2.3 MHz range was assigned to an overlapping high-frequency response of the electrolyte/cell system, including grain-boundary-like transport, dielectric relaxation,^63,75^ and possible contact/geometrical contributions, rather than to a purely isolated grain-boundary process. The associated capacitance values were in the nF range. This feature was clearly resolved up to 55 °C and became less distinguishable with increasing temperature (Figure S3), likely due to enhanced polymer mobility, increased overlap between high-frequency processes, and/or shifting of relaxation features beyond the accessible resolution. Minor inductive effects associated with cell geometry, wiring, and current-collector interfaces may also contribute to this high-frequency region.

The dominant intermediate-frequency semicircle (40 kHz–0.5 Hz) was assigned to SEI resistance, which reflects Na^+^ transport across the interphase (Figure 3b).^20,78,79^ *R_SEI_* decreased by nearly 1 order of magnitude between 30 and 50 °C, indicating strongly thermally activated ion transport across the interphase. The corresponding effective capacitance remained nearly constant (∼0.75 μF·cm^–2^). A lower-frequency feature centered near ∼13 Hz was assigned to charge-transfer processes (*R_CT_*), corresponding to interfacial Na^+^ exchange at the metal surface. In the CSPE, this process involves decomplexation of Na^+^ from the oxygen coordinating sites of the polymer chain, followed by cation reduction at the Na surface. The increasing capacitance at elevated temperature reflects enhanced interfacial activity and improved electrochemical response. At frequencies below ∼60 mHz, a diffusion-related contribution became increasingly visible with rising temperature. This response is attributed to ion transport limitations in the CSPE, consistent with the low Na^+^ transference number of PEO-based systems. However, this diffusion contribution could not be consistently resolved and reliably fitted across all temperatures.

To better resolve overlapping processes, distribution of relaxation times (DRT) analysis was performed.^80–82^ The extracted time constants (*τ*) were consistent with those obtained from equivalent circuit fitting. The DRT contour map reveals a dominant interfacial contribution at intermediate relaxation times, associated with interfacial processes, while the high-frequency region corresponds to bulk electrolyte processes (Figure 3c). SEI-related peaks (−4.3 < *log τ* < −1.5) dominated across the investigated temperature range and progressively shifted toward shorter *τ* with increasing temperature, consistent with faster interface-controlled relaxation processes at elevated temperature. In contrast, the CT (*log τ* ≈ −1.9) and diffusion-related (*log τ* > 0) processes exhibited relatively stable time constants, while their associated resistances gradually decreased with temperature, consistent with thermally activated kinetics.

In the high-frequency electrolyte-related region (*log τ* < −4.5), two distinct peaks corresponding to bulk and GB contributions were observed between 30 and 60 °C (Figure 3d). With increasing temperature, the GB-related peak shifted toward shorter relaxation times and partially overlapped with the SEI contribution. At 75 °C and above, bulk and SEI features remained distinguishable, whereas the GB contribution became less resolved.

The ionic conductivity (*σ*) of CSPE-5 was calculated from *R_B_* + *R_GB_* values and is presented as the mean ± standard deviation from three independent Na/CSPE-5/Na cells (Figure 3e). Conductivity increased with temperature, exceeding 1·10^–3^ S·cm^–1^ above 75 °C. Between 30 and 55 °C, *σ* followed an Arrhenius trend with apparent activation energy (*E_a_*) of 0.983 eV (94.8 kJ·mol^–1^, Figure S4a). This regime corresponds to semicrystalline composite polymer electrolyte, where ion transport is governed by slow segmental motion of PEO chains and cation hopping between adjacent energetically favorable oxygen coordination sites.^2,3^ Above ∼60 °C (*σ* = 6.57·10^–4^ S·cm^–1^), near the PEO melting temperature, a transition in conduction behavior toward Vogel–Tammann–Fulcher (VTF)-mechanism was observed due to enhanced segmental mobility.^83,84^

Comparative conductivity versus 1000/T analysis revealed higher apparent activation energies for the SPE (1.216 eV) and CSPE-10 (1.225 eV) relative to CSPE-5 (Figure S4a). The reduced *E_a_* of CSPE-5 indicates facilitated Na^+^ migration arising from optimized polymer-ceramic interactions that enhance segmental mobility while introducing interfacial transport pathways. In contrast, ion transport in the pristine SPE (0 wt % SBA) is governed solely by polymer segmental motion, whereas excessive filler loading (10 wt %) may restrict chain dynamics and increase tortuosity, thereby impeding effective Na^+^ conduction.^33^ Therefore, CSPE-5 was selected as the optimized composition within the investigated range of 0–10 wt % SBA, while broader filler-content optimization was beyond the scope of the present study.

Temperature-dependent SEI resistivity, presented as the mean ± standard deviation from the same three independent cells, are shown in Figure 3f. As expected, the SEI resistivity decreased markedly with temperature, from ∼32,700 Ω·cm^2^ at 30 °C to below 1,700 Ω·cm^2^ above 60 °C. This behavior is attributed to thermally enhanced ionic transport within the SEI. The SEI formed on sodium, like that on lithium, possesses a mosaic architecture composed of inorganic species (e.g., Na_2_CO_3_, NaF, Na_2_O) and organic components such as alkyl carbonates and polymeric species. Increasing temperature enhances the ionic conductivity of the SEI, particularly within the polymeric domains, facilitating ion transport across the interphase. In addition, at elevated temperatures, the SEI exhibits increased flexibility, compactness, and reduced brittleness, which improves the contact between the electrode and the solid electrolyte, thereby reducing area-specific resistance.

Compared to the SPE (0 wt % SBA), both composite electrolytes (CSPE-5 and CSPE-10) exhibited significantly lower SEI resistivity at low-temperature (Figure S4b), highlighting the beneficial role of SBA in improving interfacial contact and ionic continuity.^10,32^ Additionally, the Lewis-acidic surface of SBA may scavenge residual moisture and suppress NaTFSI hydrolysis, thereby mitigating parasitic reactions and promoting formation of a thinner, more conductive SEI.^85,86^

The transference number of sodium-ion $\left(t_{Na^{+}}\right)$ was measured by DC polarization in Na/CSPE-5/Na cells at 60 °C (Figure 4a). The current decayed to a steady-state value due to the concentration polarization. Using the modified Bruce-Vincent equation and EIS-derived resistances before and after polarization,^77,87^ the $t_{Na^{+}}$ was determined to be 0.25 ± 0.03 (N = 3, independent cells). This value is typical for PEO-based electrolytes and reflects substantial anion participation in charge transport.^36,47^

**Figure 4:**
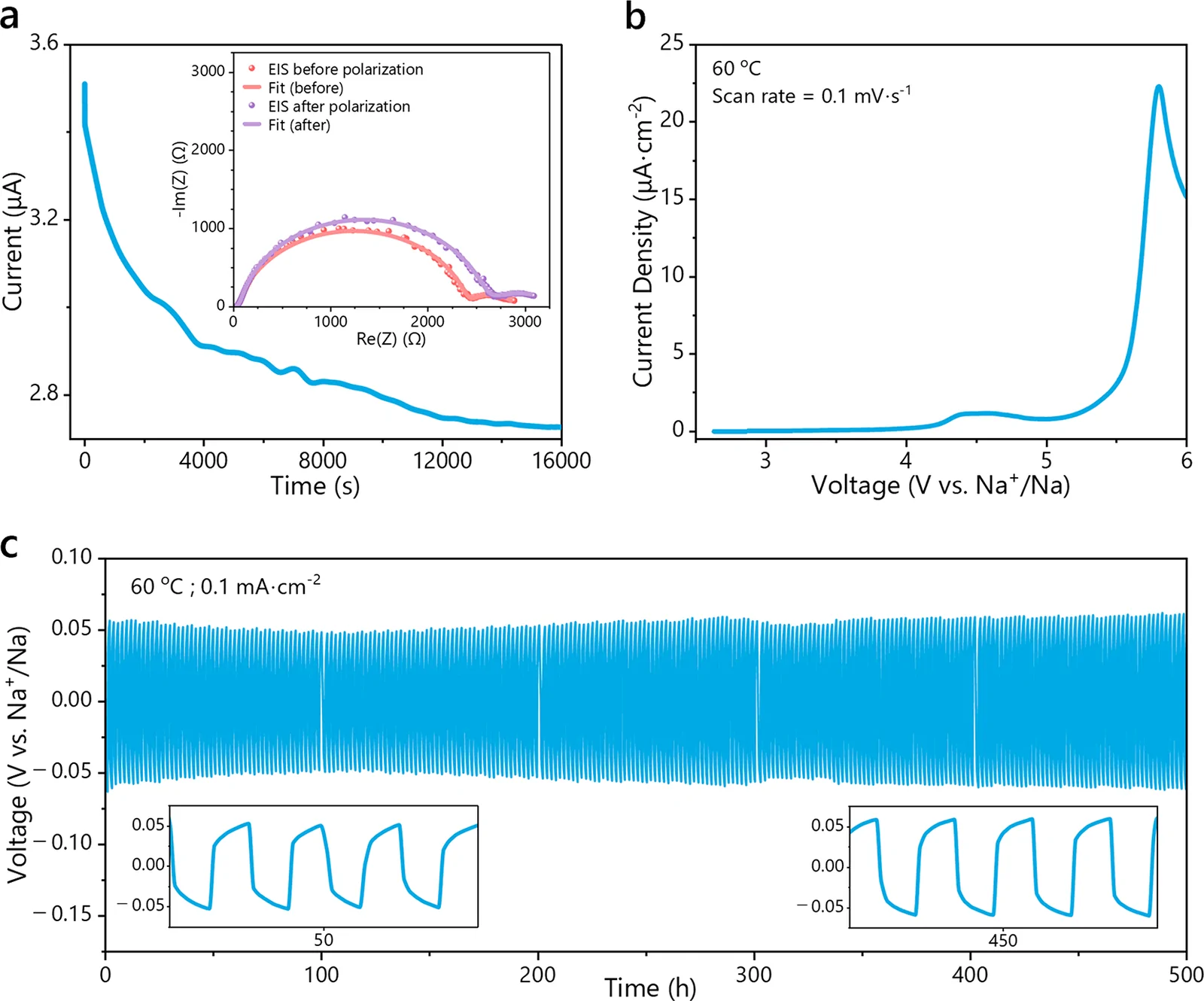
Interfacial stability and electrochemical performance of the CSPE-5. (a) DC polarization curve for Na/CSPE-5/Na cell used to determine the sodium-ion transference number $\left(t_{Na^{+}}\right)$ , with inset showing impedance spectra before and after polarization and corresponding fits. (b) LSV of SS/CSPE-5/Na cell at 60 °C (scan rate: 0.1 mV s^–1^). (c) Long-term galvanostatic cycling of Na/CSPE-5/Na cell at 0.1 mA cm^–2^ and 60 °C; insets show representative voltage profiles at early and late stages of cycling.

The linear sweep voltammetry (LSV) showed a sharp current onset beyond ∼5.0 V (vs Na^+^/Na), attributed to electrolyte decomposition (Figure 4b). Below this potential, the current remained low, indicating good oxidative stability. A minor feature at ∼4.5 V preceded the main rise and may originate from interfacial reactions, trace impurities, or early stage polymer oxidation.

Galvanostatic cycling of symmetric Na/CSPE-5/Na cell was performed at 0.1 mA·cm^–2^ (Figure 4c). Stable and symmetric voltage profiles were observed over 500 h, with minimal increase in overpotential and no evidence of short-circuiting. Early and late cycle insets show nearly identical polarization behavior, confirming a stable Na/electrolyte interface. Minor fluctuations are attributed to interfacial relaxation rather than detrimental side reactions. The stable voltage hysteresis demonstrates good Na-metal compatibility and mechanical robustness of the CSPE.

### 2.3 Structural and Chemical Characterization of the SBA–PIL Composite Interlayer

SBA–PIL composite coatings were electrophoretically deposited on NMO cathodes at 100 V using PDDA-TFSI polymerized ionic liquid as the charging agent and the binder. The deposited mass increased linearly with time up to 240 s (Figure 5a), corresponding to an average deposition rate of ∼0.7 mg·cm^–2^·min^–1^. A 60 s deposition was selected for subsequent electrochemical studies because it provided sufficient surface coverage while avoiding excessive layer thickness that could hinder Na^+^ transport, as indicated by the increased polarization observed for the 90 s coating (Figure S5).

**Figure 5:**
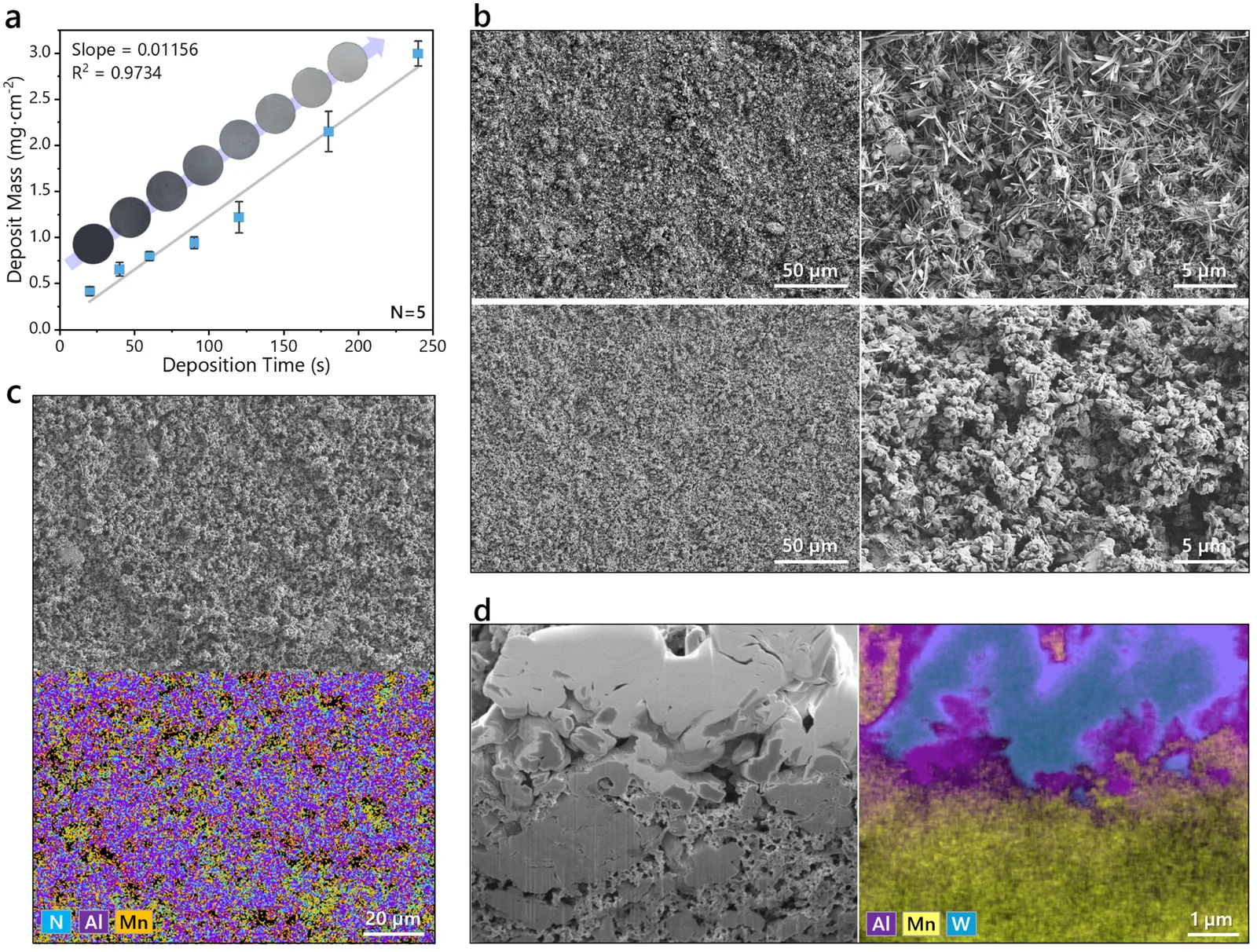
(a) Mass loading of SBA–PIL composite coatings as a function of electrophoretic deposition time at 100 V. Insets show optical images of the cathodes after deposition at different times (0–4 min), illustrating the progressive increase in coating thickness with deposition time (lighter gray appearance corresponds to thicker coatings). (b) HRSEM images of pristine (top) and after 60 s deposition (bottom) NMO cathodes. (c) Top-view SEM image and corresponding EDS elemental mapping (N, Al, Mn) of the coated cathode. (d) Cross-sectional HRSEM image and corresponding elemental mapping; The W signal arises from the protective tungsten layer sputtered prior to FIB cross-sectioning.

Figure 5b compares SEM images of pristine and coated NMO cathodes. The pristine cathode displayed a rough surface with binder visible between the active particles. After a 60 s deposition, the surface appeared smoother at low magnification, with reduced roughness indicating relative uniform coverage. At higher magnification, flake-like SBA particles (∼800 nm) were observed within the deposited layer, forming a continuous coating.

Elemental mapping shows that the Mn signal corresponds to the underlying NMO particles, while the Al signal from SBA is distributed across the surface with some localized enrichment, indicating slight aggregation of the ceramic phase (Figure 5c). Although the SBA does not form a perfectly homogeneous layer, it remains well dispersed within the coating. In contrast, the N signal from the PDDA-TFSI polymer was uniformly distributed (Figure S6). This suggests that the polymerized ionic liquid adsorbs strongly onto the surface of dispersed SBA particles in suspension, and that the electrophoretically codeposited polymer–SBA composite ultimately forms a continuous matrix covering the cathode surface. Cross-sectional SEM and EDS analysis reveal that an approximately 1.3 μm thick EPD coating, while displaying some local variations, conformally follows the cathode surface (Figure 5d). Despite some heterogeneity, the coating maintains good interfacial integrity and intimate contact with the NMO surface, without visible delamination or interfacial gaps. The composite layer appears mechanically well adhered, supporting its role as a uniform yet permeable interfacial modification rather than a dense blocking film.

XRD patterns of pristine and coated NMO cathodes are shown in Figure 6a. The pristine material was indexed to the hexagonal P2 layered structure (*P*6_3_/*mmc*). After coating, the characteristic P2 reflections were preserved, confirming structural integrity. Furthermore, lattice parameters showed negligible differences between pristine and coated samples (Table 1), indicating that the EPD process did not alter the host lattice. Secondary reflections were assigned to a birnessite-type hydrated phase (monoclinic, *C*2/*m*). This phase is electrochemically active, with a reported capacity of 83–138 mAh·g^–1^.^88^ However, since the hydrated phase is indexed in a different crystallographic setting from the P2 phase, its lattice parameters cannot be directly compared with the P2 *c*-axis spacing. Overall, the XRD results confirm that the P2 layered framework was preserved after EPD coating, with only minor changes in the secondary hydrated phase.

**Figure 6:**
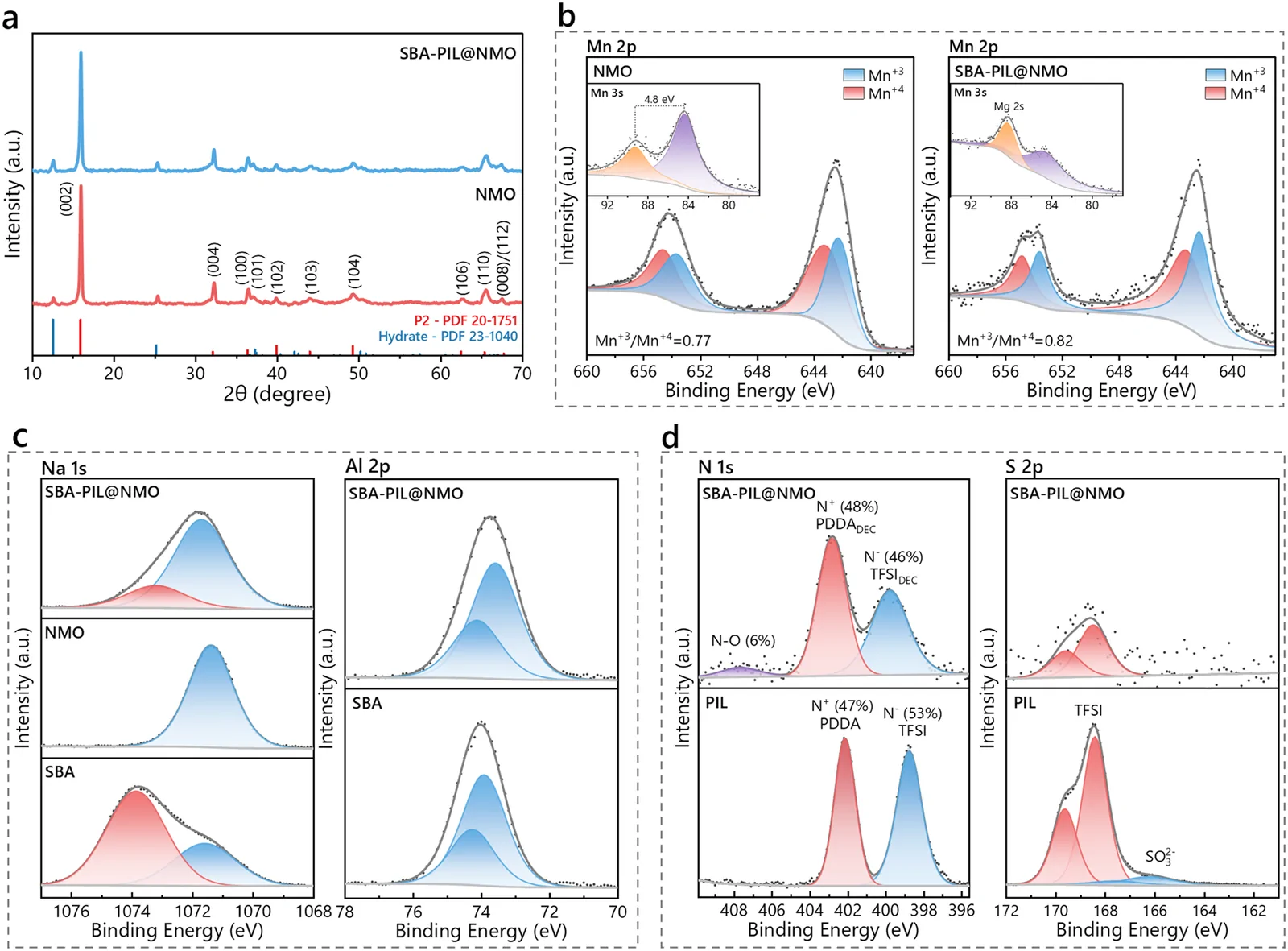
Structural and surface chemical characterization of pristine and SBA–PIL-coated NMO cathodes. (a) XRD patterns of pristine and coated cathodes. (b) High-resolution Mn 2p spectra of the cathodes, including peak deconvolution and Mn 3s insets. (c) Na 1s and Al 2p spectra of neat SBA, pristine NMO, and coated cathode. (d) N 1s and S 2p spectra of neat PIL and SBA–PIL composite coated NMO with corresponding peak fitting.

**Table 1:** Lattice Parameters of Pristine and Coated NMO Cathodes

Phase	Parameter	NMO	SBA–PIL@NMO
Sodium manganese oxide	a (Å)	2.851(1)	2.851(1)
	c (Å)	11.092(5)	11.093(5)
Sodium manganese oxide hydrate	a (Å)	4.897(1)	4.897(1)
	b (Å)	2.879(1)	2.883(1)
	c (Å)	7.228(3)	7.245(2)
	β (°)	104.00(4)	103.98(4)

The Mn 2p spectra of pristine and coated NMO exhibited characteristic Mn 2p_3/2_ (∼642.5 eV) and Mn 2p_1/2_ (∼654.2 eV) doublets (Figure 6b), deconvoluted into Mn^3+^ and Mn^4+^ contributions.^89^ The Mn^3+^/Mn^4+^ ratio changed only slightly from 0.77 (pristine) to 0.82 (coated), indicating preservation of the mixed-valence state within the uncertainty expected for surface-sensitive XPS fitting. The Mn 3s splitting further supported this conclusion, yielding an average Mn oxidation state of +3.59 for pristine NMO, corresponding to a Mn^3+^/Mn^4+^ ratio of ∼0.7 and confirming the mixed-valence nature of the layered structure. In the coated sample, quantitative analysis of the Mn 3s region was limited by overlap with the Mg 2s signal (∼88 eV),^90^ consistent with the ∼0.4 at% Mg detected for neat SBA by XPS. Taken together with the XRD results, these findings demonstrate that the EPD process successfully introduced the composite coating without altering the Mn valence distribution.

The Na 1s spectra distinguish between neat SBA, pristine NMO, and coated cathode (Figure 6c).^90^ The SBA reference exhibited a main peak at ∼1073.8 eV, assigned to Na^+^ in the β/β″-alumina conduction planes, together with a weaker contribution near ∼1071.5 eV attributed to surface Na-containing species (e.g., Na_2_CO_3_/NaOH). The pristine cathode displayed a single Na 1s component centered at ∼1071.4 eV, corresponding to lattice Na^+^ within the layered oxide framework. After coating, both Na environments were detected: the lattice signal of NMO remained dominant, while an additional higher-binding-energy feature reflected the contribution from the SBA phase. This confirms surface modification by the ceramics without altering the Na environment of the layered host structure. In addition, the Al 2p spectra of both SBA and coated cathode showed a single peak at ∼74 eV, characteristic of Al^3+^ in an oxide framework,^90,91^ indicating that Al oxidation state maintains during deposition.

On the contrary, the N 1s and S 2p spectra indicate substantial TFSI transformation during coating process (Figure 6d).^57,92^ In the neat PIL, the N 1s spectrum displayed two well-resolved components at 402.2 eV (N^+^ from PDDA) and 398.7 eV (imide N^–^ from TFSI), with a cation-to-anion area ratio of 47:53, consistent with the expected stoichiometry. After deposition, the cation peak shifted slightly to 402.8 eV, while the anionic N peak broadened and shifted to ∼399.8 eV, indicative of TFSI decomposition into imide/sulfonamide-type fragments. An additional weak feature appeared at ∼407.8 eV (∼6%) and was assigned to nitrate-type N–O_*x*_ species (e.g., NaNO_3_), possibly formed from oxidized TFSI-derived fragments during deposition. The S 2p signal in the coated sample was markedly attenuated compared to neat PIL, and the characteristic intense TFSI doublet at ∼168.5 eV was no longer clearly resolved. Low-intensity contributions remained in the 167–169 eV region, indicating that sulfur-containing species persist but their concentration is significantly reduced (∼0.06 at%) and partially transformed during EPD. Overall, the PDDA cation was largely retained in the deposited layer, whereas the TFSI-related signal was strongly attenuated and partially transformed during deposition. Quantitative XPS analysis indicated an SBA-to-PIL molar ratio of ∼1:1.3, corresponding to ∼14 wt % PDDA binder within the composite coating.

### 2.4 Electrochemical Performance and Interfacial Stabilization in PEO-Based CSPE Cells

The rate capability of pristine and SBA–PIL coated NMO cathodes was evaluated at 60 °C in the PEO-based CSPE-5 system (Figure 7a). During initial C/40 formation cycles (CC–CV), the pristine cathode delivered higher capacity. In the subsequent C/40 step (CC), the capacity gap remained pronounced (∼95 vs ∼88 mAh g^–1^), consistent with a modest increase in interfacial resistance or a slight reduction in effective active area in the coated sample under low-rate conditions. As current density increased, the capacity difference diminished, and at the highest rate the coated cathode retained slightly higher capacity, indicating reduced polarization under practical operating currents. Upon returning to C/30, the coated cathode showed superior capacity recovery, evidencing improved interfacial robustness under high-rate stress. The rate-dependent capacity decrease arises from the combined effect of intrinsically slower Na^+^ diffusion kinetics in layered sodium cathodes and transport limitations in the PEO-based CSPE, including concentration polarization associated with its low transference number.

**Figure 7:**
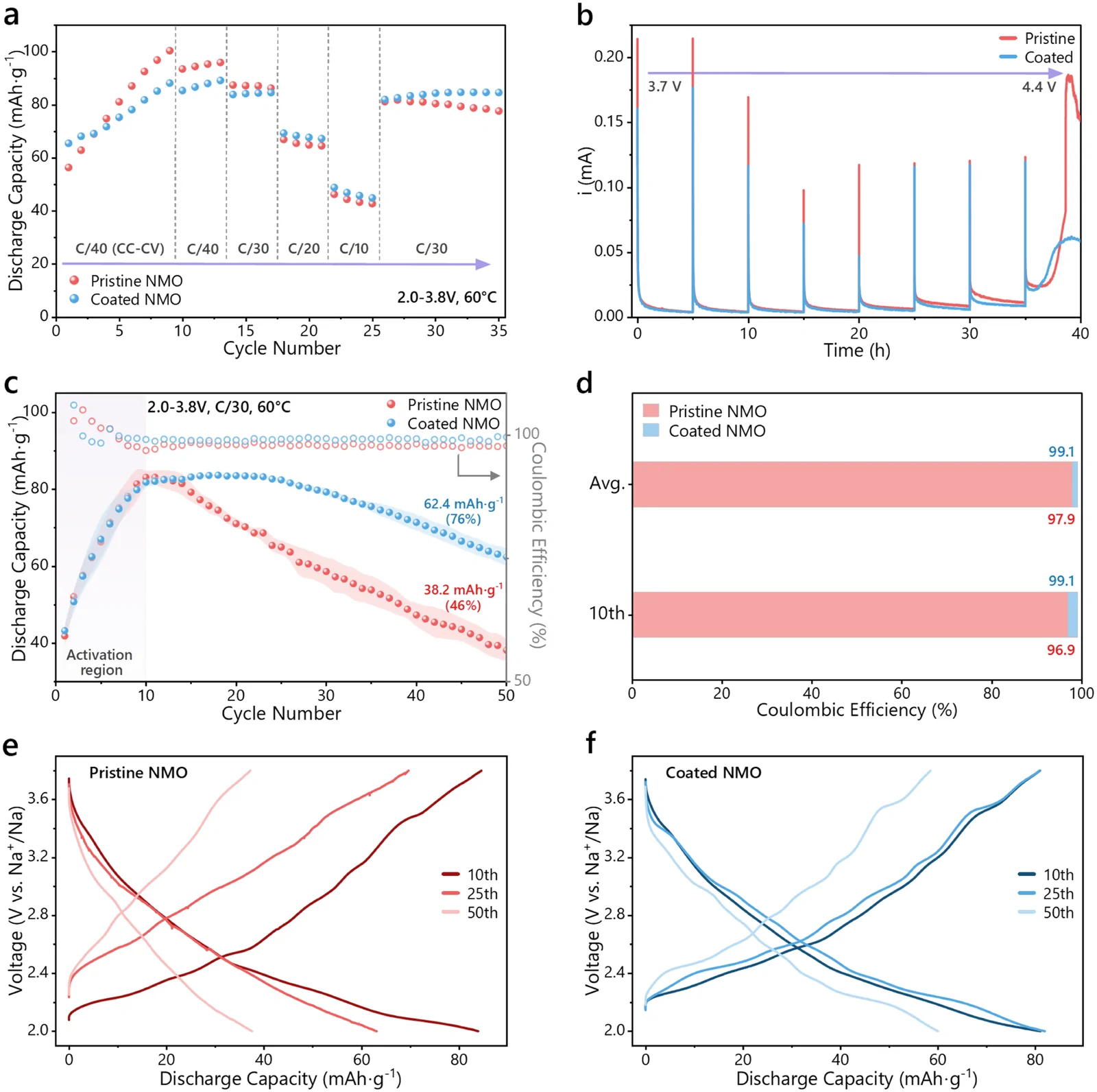
Electrochemical performance of pristine and coated NMO in NMO/CSPE/Na half-cells at 60 °C. (a) Rate capability from C/40 to C/10 and recovery at C/30 within 2.0–3.8 V. (b) Chronoamperometric voltage-step response from 3.7 to 4.4 V (0.1 V increments, 5 h hold per step). (c) Long-term cycling performance at C/30 (2.0–3.8 V) and corresponding Coulombic efficiency. The capacity values are presented as mean ± standard deviation from three independent cells. (d) 10th-cycle and average Coulombic efficiency comparison calculated from three independent cells. Charge–discharge profiles of (e) pristine and (f) coated NMO at the 10th, 25th, and 50th cycles.

Chronoamperometric voltage-step experiments (ΔV = 0.1 V, 5 h holds) were conducted on preactivated NMO/Na cells (Figure 7b). In the lower voltage range, the coated cathode exhibited slightly reduced initial current spikes, indicating a modest increase in high-frequency resistance. However, current relaxation behavior was similar for both cathodes, suggesting that under moderate polarization the interlayer did not significantly modify the longer-time interfacial or diffusion-controlled processes. Above 4.2 V, a divergence between the two cathodes became apparent, as the pristine cathode exhibited systematically higher hold currents. This behavior indicated enhanced parasitic reactivity and reduced interfacial properties of the surface. At 4.4 V, a pronounced current rise was observed, characteristic of accelerated oxidative electrolyte decomposition. This increase was clearly suppressed in the coated cathode, demonstrating improved oxidative stability under elevated polarization.

Long-term cycling was performed at C/30 because higher current rates are strongly affected by transport limitations in PEO-based sodium solid polymer cells, as reflected by the rate-capability results. This rate was therefore selected to provide sufficient accessible capacity while enabling evaluation of polarization growth and interface-driven degradation. Galvanostatic cycling of NMO/Na cells revealed a progressive activation period during the first ∼10 cycles, which may involve improved interfacial contact, polymer softening, interphase reconstruction, and gradual access to previously less accessible active particles (Figure 7c). After stabilization, the coated cathodes retained 76% of their cycle-10 capacity at cycle 50, compared with 46% for pristine NMO, delivering 62.4 mAh g^–1^ and 38.2 mAh g^–1^, respectively. Coulombic efficiency (CE) further supported the stabilizing effect of the composite interlayer (Figure 7d). At cycle 10, the coated cells exhibited a CE of 99.1%, compared with 96.9% for pristine NMO. Over cycles, the average CE remained 99.1% (cycles 10–50) for the coated cells compared with 97.9% for pristine, indicating reduced parasitic charge consumption and improved interfacial stability.

For comparison, cells assembled with a SPE (0 wt % SBA) were cycled under identical conditions (Figure S7). The SPE cells delivered higher and initially stable capacity, indicating improved initial interfacial contact and/or lower polarization. However, the capacity subsequently decreased sharply, and the cell short-circuited before 40 cycles. In contrast, the CSPE-based cells maintained stable operation throughout the test. This behavior suggests that ceramic-filled composite electrolyte enhances interfacial robustness of Na/NMO cells and mitigates premature short-circuiting, likely by promoting more uniform Na deposition and improving mechanical resistance to dendrite penetration.

Charge/discharge profiles showed comparable behavior at cycle 10 following activation (Figure 7e, f). The coated cathode exhibited improved plateau definition by cycle 25, indicating progressive interfacial stabilization, whereas the pristine electrode showed gradual polarization growth. By cycle 50, the coated cathode maintained its profile with moderate hysteresis increase, while the pristine cathode exhibited pronounced hysteresis expansion and slope distortion, consistent with increased interfacial resistance and reduced accessible capacity.

To elucidate the origin of the distinct cycling behavior of pristine and SBA–PIL coated NMO cathode, EIS measurements were performed at selected cycle numbers during galvanostatic cycling. Spectra were collected after discharge to 2.0 V, while the as-assembled cells measured at open-circuit voltage are presented as a baseline reference (Figure S8). Representative Nyquist plots collected at the 10th cycle following activation, at the 20th cycle where the divergence in electrochemical stability was most evident, and at the 50th cycle, are presented (Figure 8a). The spectra were fitted using an *R*-*Q* equivalent circuit to separate the effective series resistance *R_B_*, the electrode–electrolyte interphase resistance *R*_*EEI*_, and the charge-transfer resistance *R_CT_* (Figure 8b).^76,93^ A finite-length Warburg element was included to describe low-frequency diffusion, although this contribution was not clearly resolved in all spectra. Complete fitting parameters are provided in the Supporting Information (Table S2).

**Figure 8:**
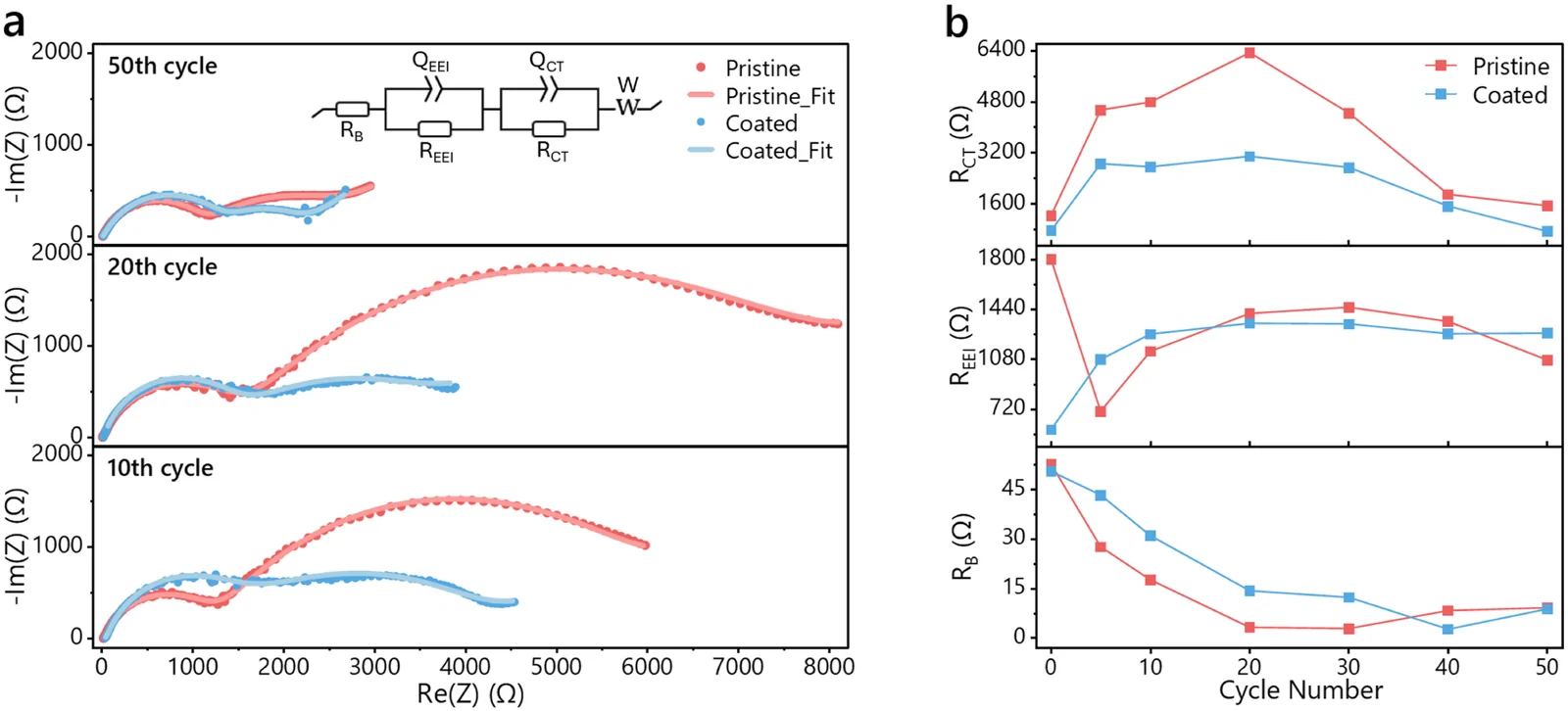
Impedance evolution of pristine and coated NMO in NMO/Na half-cells at 60 °C. (a) Nyquist plots recorded at the 10th, 20th, and 50th cycles, together with equivalent circuit fits (inset). (b) Evolution of the fitted resistance components as a function of cycle number: bulk resistance (*R_B_*), electrode–electrolyte interphases resistance (*R*_EEI_), and charge-transfer resistance (*R_CT_*).

Both cells exhibited similar initial *R_B_* values (∼50–53 Ω), indicating comparable baseline ohmic resistance. Upon cycling, *R_B_* decreased in both systems, suggesting progressive stabilization during the initial cycling stages.

More distinct differences were observed in the interfacial components. The pristine cathode exhibited a high initial *R_EEI_* (1799 Ω), whereas the coated cathode showed a substantially lower value (572 Ω). *R_EEI_* value includes the contribution of anode/electrolyte and cathode/electrolyte interphase resistances. Although the composite interlayer introduces an additional surface layer, the substantially lower initial *R_EEI_* indicates that it did not hinder interfacial ionic transport. Instead, it suggests that the interlayer is highly compatible with composite electrolyte and, it promotes formation of a more ionically accessible and chemically stabilized EEI. With cycling, *R_EEI_* in both cells converged to comparable magnitudes, although the coated electrode displayed a more gradual and controlled evolution.

The most pronounced distinction was observed in the charge-transfer resistance. *R_CT_* increased during early cycles in both cathodes. For the coated cathode, *R_CT_* rose to ∼3.1 kΩ and remained relatively stable between cycles 5 and 30. In contrast, the pristine cathode exhibited consistently higher *R_CT_* values and continued to increase beyond cycle 5, reaching a maximum of ∼ 6.3 kΩ at cycle 20. At extended cycling, *R_CT_* decreased in both systems, likely reflecting ongoing structural and interfacial evolution. Notably, the coated cathode maintained lower *R_CT_* throughout cycling, indicating moderate kinetic deterioration. A Warburg-type diffusion response was more clearly resolved when *R_CT_* was comparatively low and was therefore predominantly observed in the coated cathode. In contrast, during intermediate cycling of the pristine cathode, where *R_CT_* was high, the diffusion contribution became masked by the dominant interfacial kinetic resistance. In summary, the impedance findings demonstrate that the SBA–PIL interlayer regulates evolution of cathode/CSPE interfacial resistance and mitigates kinetic deterioration during cycling, which correlates with the improved capacity retention and reduced polarization of the coated NMO cathode.

## 3 Conclusions

An SBA–PIL composite interlayer was introduced onto layered Na_0.7_MnO_2_ cathodes via electrophoretic deposition, forming a conformal yet permeable surface modification while maintaining the P2 crystal structure and Mn mixed-valence state. Structural and spectroscopic analyses confirmed a continuous PDDA-based matrix embedding dispersed SBA particles, establishing an ionically accessible and chemically modified surface interface.

The ceramic-filled PEO-based CSPE provided a mechanically reinforced Na electrolyte with ionic conductivity comparable to the SPE at 60 °C, but with lower activation energy for Na^+^ transport (0.983 eV vs 1.216 eV). Although bulk conductivity was similar at the operating temperature, SBA incorporation improved electrolyte stabilization by reinforcing the polymer matrix and preventing premature short-circuiting observed in SPE cells. These results highlight that the beneficial role of the ceramic filler is not limited to bulk ion transport, but also involves improved mechanical integrity and more stable interface-controlled kinetics. Nevertheless, layered NMO cathodes still exhibited progressive interfacial resistance growth and kinetic deterioration.

The composite interlayer effectively mitigated these limitations. Coated cathodes showed improved rate recovery, suppressed high-voltage parasitic currents, higher Coulombic efficiency (99.1% vs 97.9%), and enhanced capacity retention (76% vs 46% after 50 cycles). Voltage-capacity profiles showed reduced polarization growth, while impedance analysis showed moderated evolution of interphase and charge-transfer resistances, indicating more controlled interfacial kinetics.

Although cycling stability remains limited by intrinsic Na^+^ transport constraints and the oxidative stability of PEO-based electrolytes, this study demonstrates that composite interlayer engineering effectively regulates the cathode-electrolyte interface and stabilizes charge-transfer behavior in sodium metal solid polymer batteries. Overall, the work establishes a proof-of-concept framework for combining electrolyte-level mechanical reinforcement with cathode-side interfacial regulation to stabilize layered oxide cathodes in PEO-based sodium metal batteries.

## 4 Experimental Section

### 4.1 Preparation of SPE and CSPE Films

Solid polymer electrolyte (SPE) and composite solid polymer electrolytes (CSPEs) were prepared inside an argon-filled glovebox using anhydrous acetonitrile (AcroSeal, Thermo Scientific Chemicals) as the solvent. Poly­(ethylene oxide) (PEO, *M*_w_ = 600,000 g·mol^–1^, Thermo Scientific) and sodium bis­(trifluoromethanesulfonyl)­imide (NaTFSI, Solvionic) were dissolved in dry acetonitrile at an EO:NaTFSI molar ratio of 18:1 under continuous stirring until a homogeneous, clear solution was obtained. For comparison, a ceramic-free solid polymer electrolyte, denoted as SPE, was prepared using the same PEO:NaTFSI ratio without SBA filler. For composite electrolytes, sodium-β″-alumina powder (SBA, NaAl_5_O_8_ with Mg dopant, MSE Supplies) was added to the polymer solution to yield ceramic filler contents of 5 and 10 wt %, denoted as CSPE-5 and CSPE-10, respectively. SEM analysis showed that the SBA powder consisted of irregular flake-like/platelet particles. Representative flakes had an approximate lateral length of ∼800 nm and thickness of ∼40 nm. The resulting polymer suspensions were cast into Teflon molds, and solvent evaporation was carried out in a vacuum desiccator at 60 °C until free-standing films were formed. The dried films were cut into 17 mm-diameter discs and further dried under vacuum at 50 °C prior to cell assembly.

### 4.2 Electrophoretic Deposition of SBA–PIL Composite Interlayers

Neat sodium-β″-alumina (SBA, NaAl_5_O_8_ with Mg dopant, MSE Supplies) was deposited onto layered-P2-type sodium manganese oxide cathodes (Na_0.7_MnO_2_, NMO, NEI Corporation; active material loading: 6–8 mg·cm^–2^, 0.9 ± 0.04 mAh·cm^–2^) via cathodic electrophoretic deposition (EPD). Polydiallyldimethylammonium bis­(trifluoromethanesulfonyl)­imide (PDDA-TFSI, Solvionic), a polymerized ionic liquid (PIL), was employed as both a charging agent for particle migration and a binder within the deposited composite layer. The EPD suspension was prepared in anhydrous acetonitrile (Fisher Scientific) containing SBA and PIL at a 60:40 wt % ratio, with the addition of 0.016% (w/v) Triton X-100 (Sigma-Aldrich), resulting in a total solid concentration of 0.55 mg·mL^–1^. The suspension was homogenized prior to deposition. A constant voltage of 100 V was applied between the NMO cathode sheet and a carbon counter electrode using a Keithley 2611B SourceMeter. After deposition, the coated cathodes were punched into 10 mm-diameter discs and dried under vacuum at 100 °C overnight. All coated electrodes were subsequently transferred to and stored in an argon-filled glovebox.

### 4.3 Structural and Surface Characterizations

The morphologies of the polymer electrolyte films and cathodes surfaces were examined using a high-resolution field emission scanning electron microscope (HRSEM, Zeiss GeminiSEM 300). Energy-dispersive X-ray spectroscopy (EDS) was performed using a Bruker X-Flash 6/60 detector. Electrolyte samples were sputter-coated with a ∼5 nm chromium layer prior to imaging to minimize charging effects. Cross-sectional imaging of coated cathode was conducted using a focused ion beam scanning electron microscopy (FIB-SEM) system (Helios 5 UC DualBeam, Thermo Scientific) following tungsten deposition. Phase and structural analyses of SBA powder, neat PEO, polymer electrolyte films, and NMO cathodes were performed by X-ray diffraction (XRD, Bruker D8 Discover) using Cu Kα radiation (1.5406 Å) with a step size of 0.02°. Attenuated total reflectance Fourier-transform infrared (ATR-FTIR) spectra were collected using a Bruker Tensor 27 spectrometer over the range 4000–400 cm^–1^ at a resolution of 4 cm^–1^. X-ray photoelectron spectroscopy (XPS) measurements were carried out using a Thermo Scientific ESCALAB QXi XPS Microprobe equipped with a monochromatic Al Kα source (400 μm spot size, 0.1 eV energy resolution). Samples were mounted under argon atmosphere and transferred to the analysis chamber without air exposure.

### 4.4 Electrochemical Characterization

CR2032-type coin cells were assembled in an argon-filled glovebox (H_2_O and O_2_ < 2 ppm). Sodium metal discs (12 mm diameter, AOT Battery), laminated onto aluminum foil, were used as the anode in half-cells. Layered P2-type Na_0.7_MnO_2_ cathodes (10 mm diameter; loading: 6–8 mg·cm^–2^, 0.9 ± 0.04 mAh·cm^–2^) and 17 mm solid electrolyte films were employed.

Temperature-dependent ionic conductivity and interfacial resistances were evaluated by electrochemical impedance spectroscopy (EIS) using Na/SPE/Na symmetric cells over a temperature range of 30–90 °C. Measurements were conducted with a BioLogic SP-300 potentiostat in the frequency range from 7 MHz to 10 mHz using an AC perturbation of 20 mV. Equivalent circuit fitting was performed using BioLogic Zfit software, and distribution of relaxation times (DRT) analysis was carried out using pyDRTtools.^94,95^ Sodium-ion conductivity, activation energy, and transference number were determined from symmetric cells following established procedures.^77,87^ The ionic conductivity (*σ*) was calculated using

1 $$\sigma =\frac{l}{R_{Ele}\cdot A}$$

where *l* is the electrolyte thickness, *A* is the electrode–electrolyte interfacial area, and *R_Ele_* is the total electrolyte resistance obtained from EIS fitting (*R_B_* + *R_GB_*). The temperature dependence of conductivity followed an Arrhenius-type relation:

2 $$\sigma =\sigma_{0}\cdot \exp\left(-\frac{E_{a}}{RT}\right)$$

where *E_a_* is the activation energy, *R* is the gas constant, and *T* is the absolute temperature. Activation energies were extracted from the slope of *ln*(*σ*) versus 1000/T plots.

All subsequent electrochemical measurements were conducted at 60 °C. Sodium-ion transference numbers $\left(t_{Na^{+}}\right)$ were determined using combined EIS and DC polarization (10 mV) according to the Bruce-Vincent method:

3 $$t_{Na^{+}}=\frac{i_{ss}\left(\Delta V-i_{0}R_{int,0}\right)}{i_{0}\left(\Delta V-i_{ss}R_{int,ss}\right)}$$

Where *i*_0_ and *i_ss_* are the initial and steady-state currents, and *R_int_*,_0_ and *R_int_*,*_ss_* are the interfacial resistances before and after polarization, respectively. Electrochemical stability was assessed by linear sweep voltammetry (LSV) using stainless-steel/SPE/Na cells from open-circuit voltage to 6.0 V vs Na^+^/Na at a scan rate of 0.1 mV·s^–1^. Galvanostatic cycling of Na/SPE/Na symmetric cells was performed at a current density of 0.1 mA·cm^–2^.

All electrochemical analyses for evaluation composite interlayer performance were conducted on NMO/CSPE/Na half-cells at 60 °C. Rate capability tests were performed within a voltage window of 2.0–3.8 V. Cells were initially activated at C/40 using a constant-current constant-voltage (CC–CV) charging protocol, including a 3 h hold at the upper cutoff voltage. Following activation, rate capability tests were performed sequentially at C/40, C/30, C/20, and C/10, and finally returned to C/30 to evaluate capacity recovery. Chronoamperometric voltage-step tests were conducted after ten formation cycles with a cutoff voltage of 3.7 V. Sequential voltage holds were applied from 3.7 to 4.4 V in 0.1 V increments, with a 5 h hold at each potential. Galvanostatic charge–discharge cycling was performed at C/30 within a voltage window of 2.0–3.8 V using a BioLogic VSP-3e potentiostat. During activation, a constant current constant voltage (CC–CV) charging protocol was applied, with a 3 h hold at the upper cutoff voltage. EIS measurements were performed at open-circuit voltage for the as-assembled cells after 24 h equilibrium at 60 °C. Spectra were collected over a frequency range of 1 MHz to 20 mHz using a 20 mV perturbation. For cycling-dependent comparison, EIS measurements were periodically performed during long-term cycling after discharge to 2.0 V followed by a 1 h rest prior to measurement.

## Supplementary Material



## Notes

HRSEM images of SPE, CSPE-5, and CSPE-10; XRD analysis of SBA; EIS fitting parameters; additional Nyquist plots and Arrhenius analysis; comparative SEI resistivity; voltage profiles of SBA–PIL coatings with different deposition times; SEM and EDS elemental mapping of the coated cathode; cycling comparison of SPE and CSPE; impedance spectra of as-assembled cells; equivalent-circuit fitting parameters for cycled cells (PDF)
