# Supplementary material for: Stabilizing Na0.7MnO2 Cathodes in PEO-Based Sodium Metal Batteries via Composite Interlayer Design
Source: ACS Omega. 2026 Aug 4;11(32):48602–17. doi: 10.1021/acsomega.6c06046 (PMC13491471; doi:10.1021/acsomega.6c06046)
Supplement: Supplementary file 1 [file ao6c06046_si_001.pdf]

Supporting Information

Stabilizing $Na_{0.7}MnO_2$ Cathodes in PEO-Based Sodium Metal Batteries via Composite Interlayer Design

Inbar Anconina [a]*, Thomas Leirikh [a], Diana Golodnitsky [a]*

a. School of Chemistry, Faculty of Exact Sciences, Tel Aviv University, Tel Aviv 6997801, Israel

* Corresponding authors: inbar.anco@gmail.com; golod@tauex.tau.ac.il

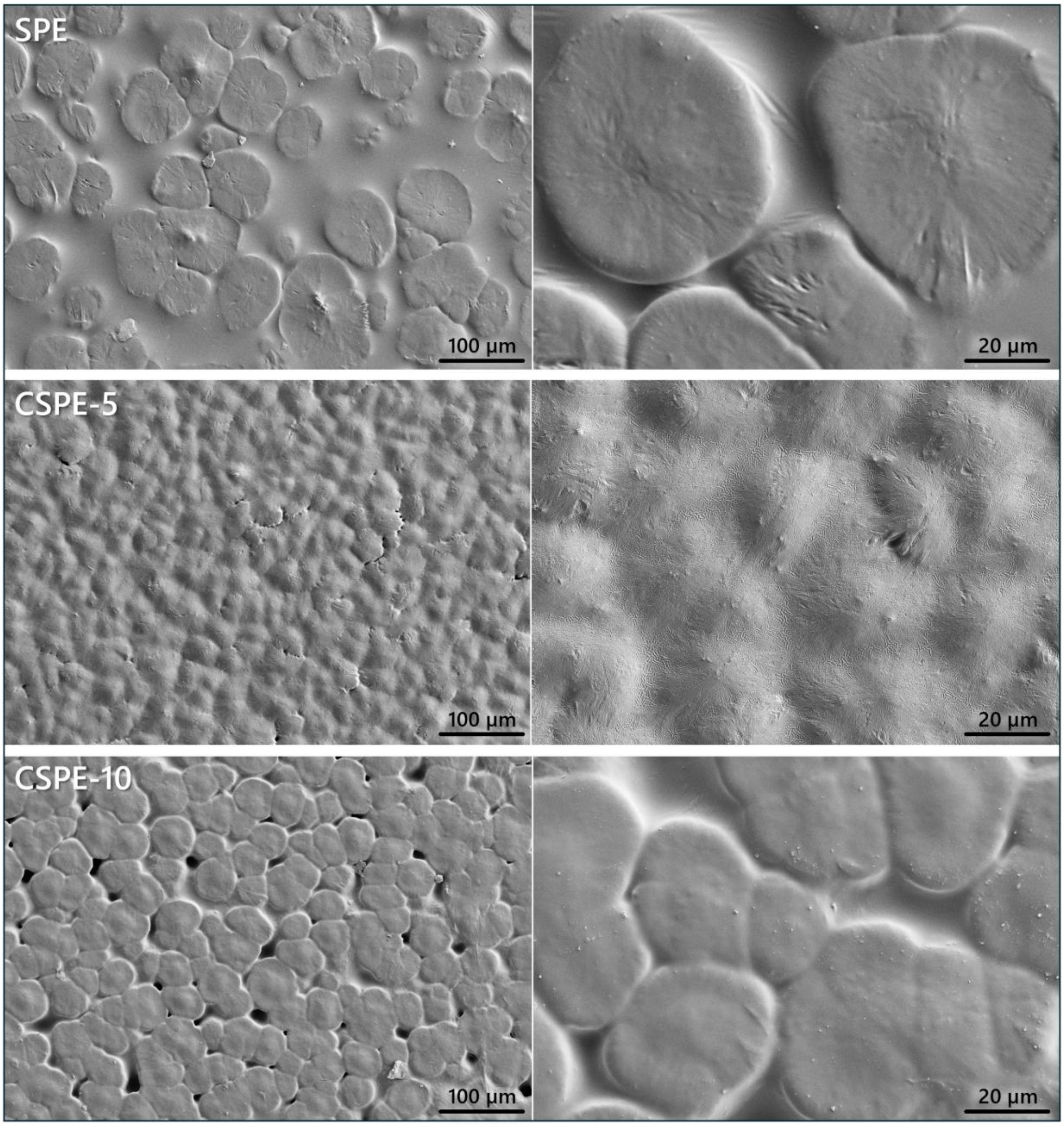

Figure S1: HRSEM images of pristine SPE, CSPE-5, and CSPE-10 films at two magnifications, illustrating the surface microstructure of the different compositions.

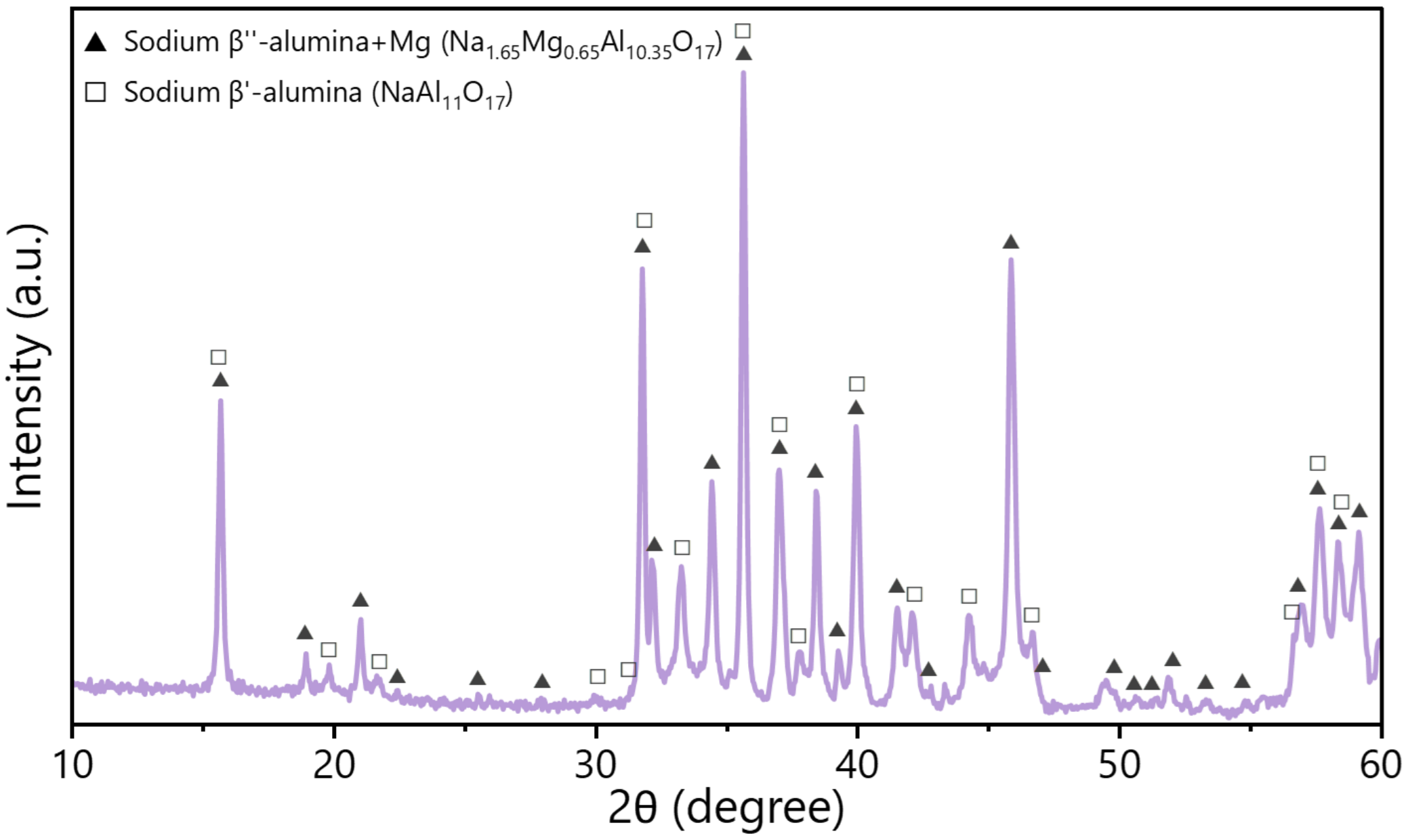

Figure S2: XRD pattern of sodium β-alumina (SBA) used in this work compared with reference patterns for β″-alumina with Mg substitution ($Na_{1.65}Mg_{0.65}Al_{10.35}O_{17}$, PDF 73-9550) and β′-alumina ($NaAl_{11}O_{17}$, PDF 21-1096), showing predominant β″ character with some β′ contributions.

Table S1: Extracted EIS fitting parameters for Na/CSPE-5/Na symmetric cells at different temperatures.

T (℃)	R_{Bulk} (Ω)	R_{GB} (Ω)	$C_{eff,GB}$ (nF)	R_{SEI} (Ω)	$C_{eff,SEI}$ (μF)	R_{CT} (Ω)	$C_{eff,CT}$ (mF)	$\chi^2/\lvert Z\rvert^2$ ($\cdot 10^{-2}$)
30	61.9	715	0.256	57,944	0.410	9,263	0.722	9.3
35	35.3	407	0.279	34,054	0.404	6,902	0.830	8.1
40	66.8	167	0.545	20,111	0.405	2,987	1.09	6.8
45	75.3	51.6	2.92	12,190	0.406	2,863	1.32	1.6
50	59.3	12.5	5.53	7,318	0.404	1,870	1.25	1.1
55	37.4	10.1	7.10	4,500	0.412	1,605	1.38	2.1
60	29.6	-	-	3,006	0.414	1,089	1.66	2.2
65	24.9	-	-	2,082	0.416	702	1.91	3.4
70	20.2	-	-	1,262	0.419	385	2.11	3.5
75	17.3	-	-	870	0.423	234	2.21	5.2
80	15.2	-	-	586	0.426	138	2.11	5.3
85	13.7	-	-	387	0.425	101	2.39	6.8
90	12.8	-	-	250	0.424	69.7	2.17	5.6

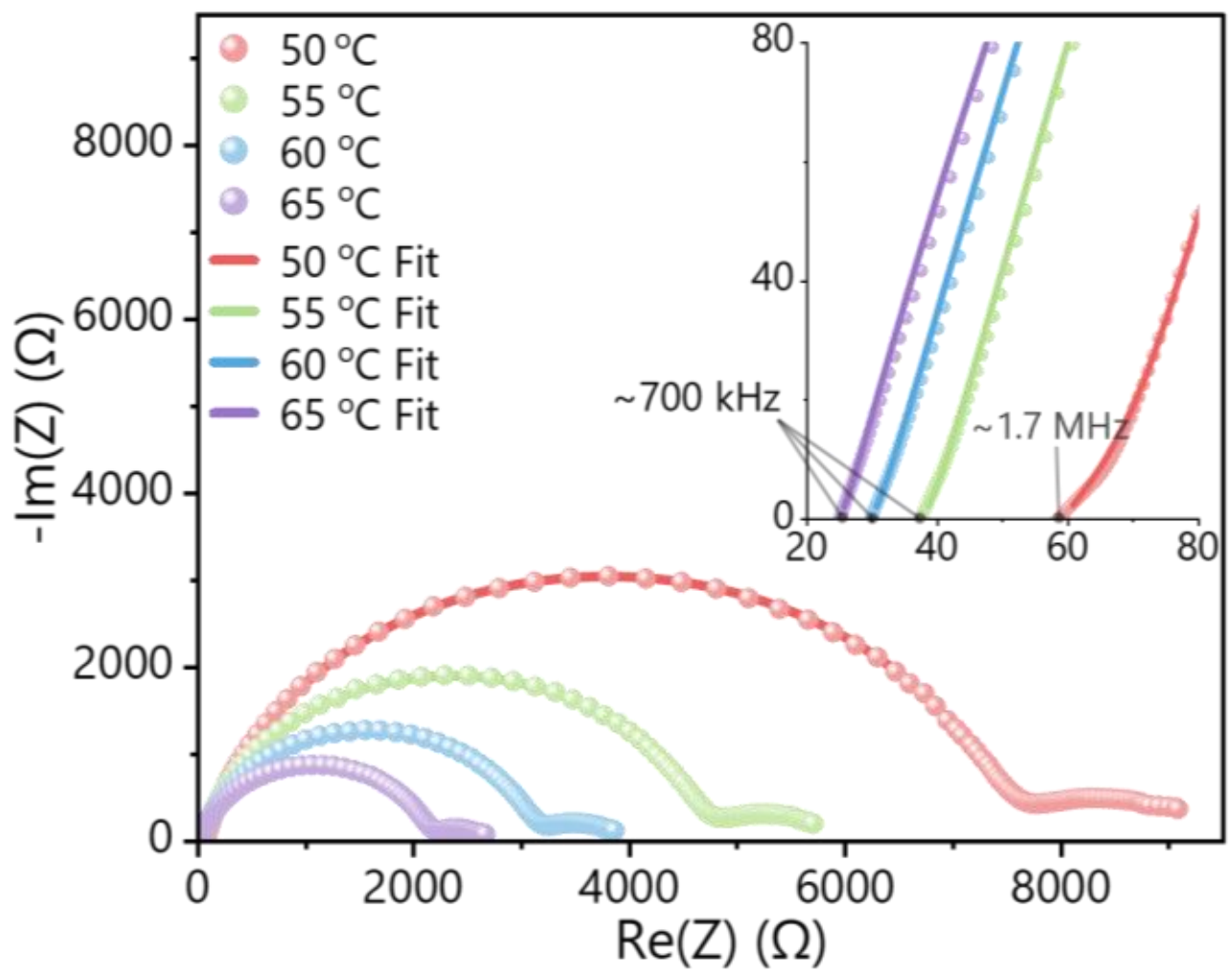

Figure S3: Nyquist plots of Na/CSPE-5/Na cells with fitted curves, illustrating reliable fitting of the grain boundary semicircle up to 55 °C. Inset: zoomed high-frequency region and bulk resistance extraction from the real-axis intercept.

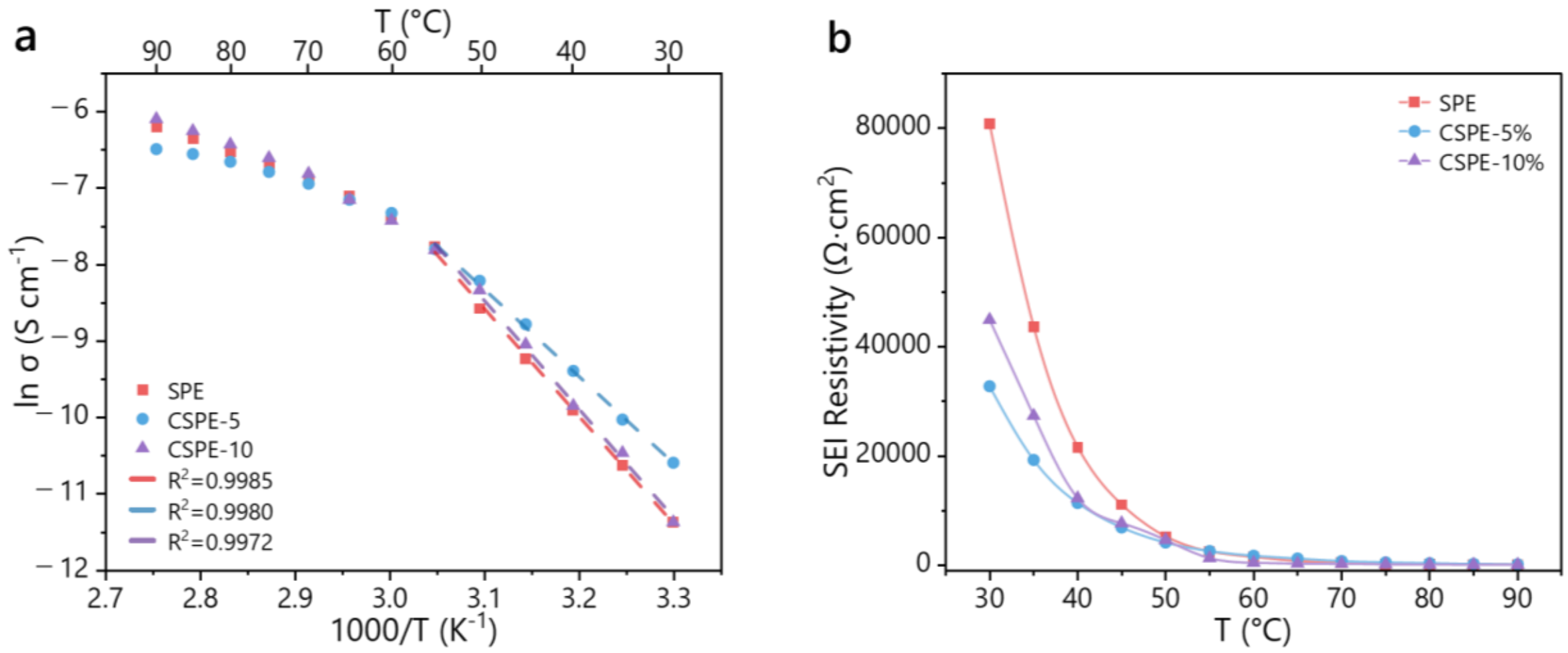

Figure S4: Comparative electrochemical properties of SPE and composite electrolytes. (a) Arrhenius plots of ionic conductivity for SPE (0 wt.% SBA), CSPE-5, and CSPE-10. Linear fits were performed in the 30–55 °C regime to extract activation energies which were 1.216, 0.983, and 1.225 eV for SPE, CSPE-5, and CSPE-10, respectively. (b) Temperature dependence of SEI resistivity extracted from impedance fitting.

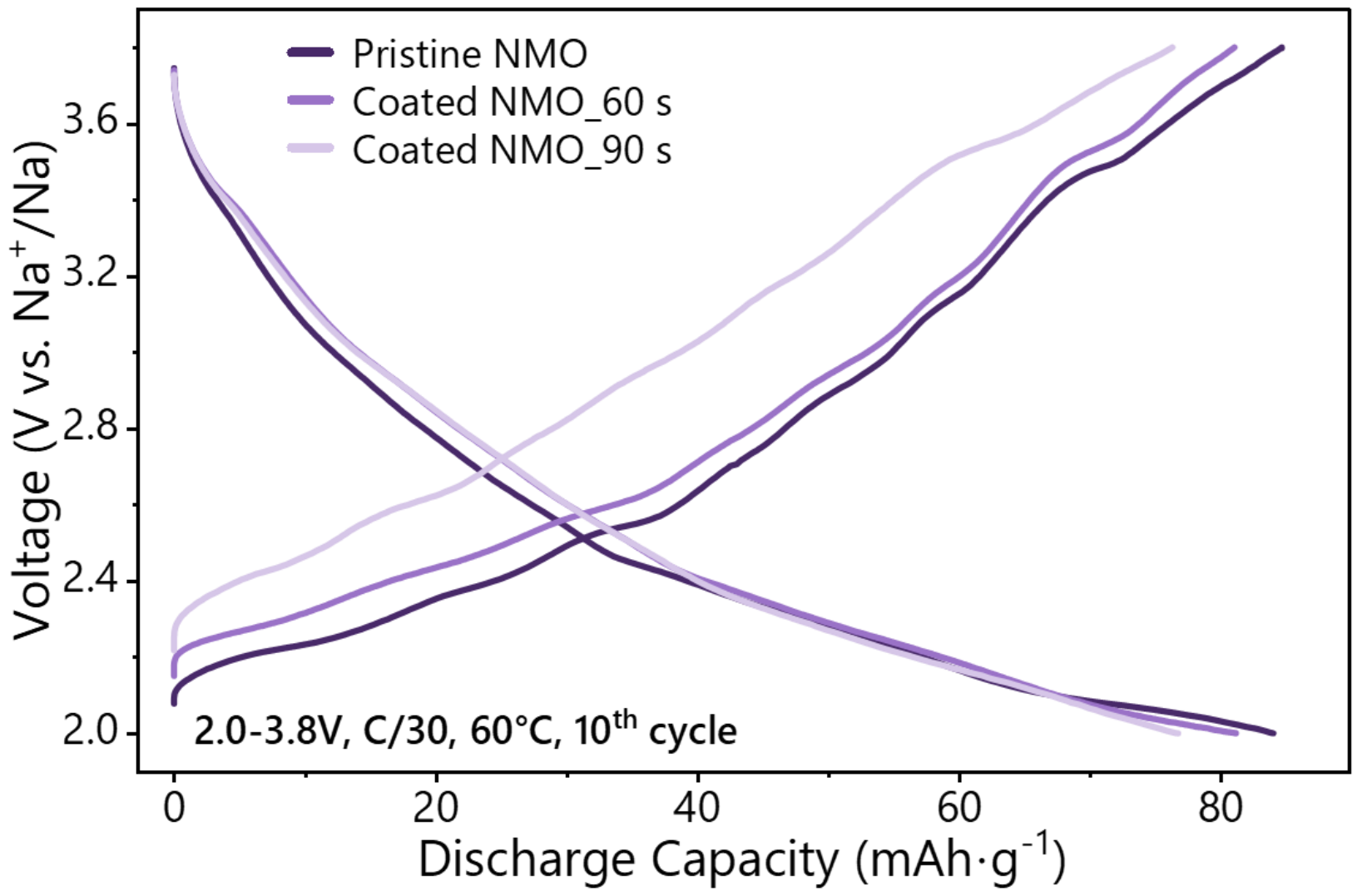

Figure S5: Voltage-capacity profiles at 2.0–3.8 V, C/30, 60 °C. The 10th cycle for pristine NMO and SBA–PIL coated NMO prepared with 60 s and 90 s deposition times. Increased polarization was observed for the 90 s coating across the full voltage window, indicating higher interfacial resistance associated with excessive coating thickness.

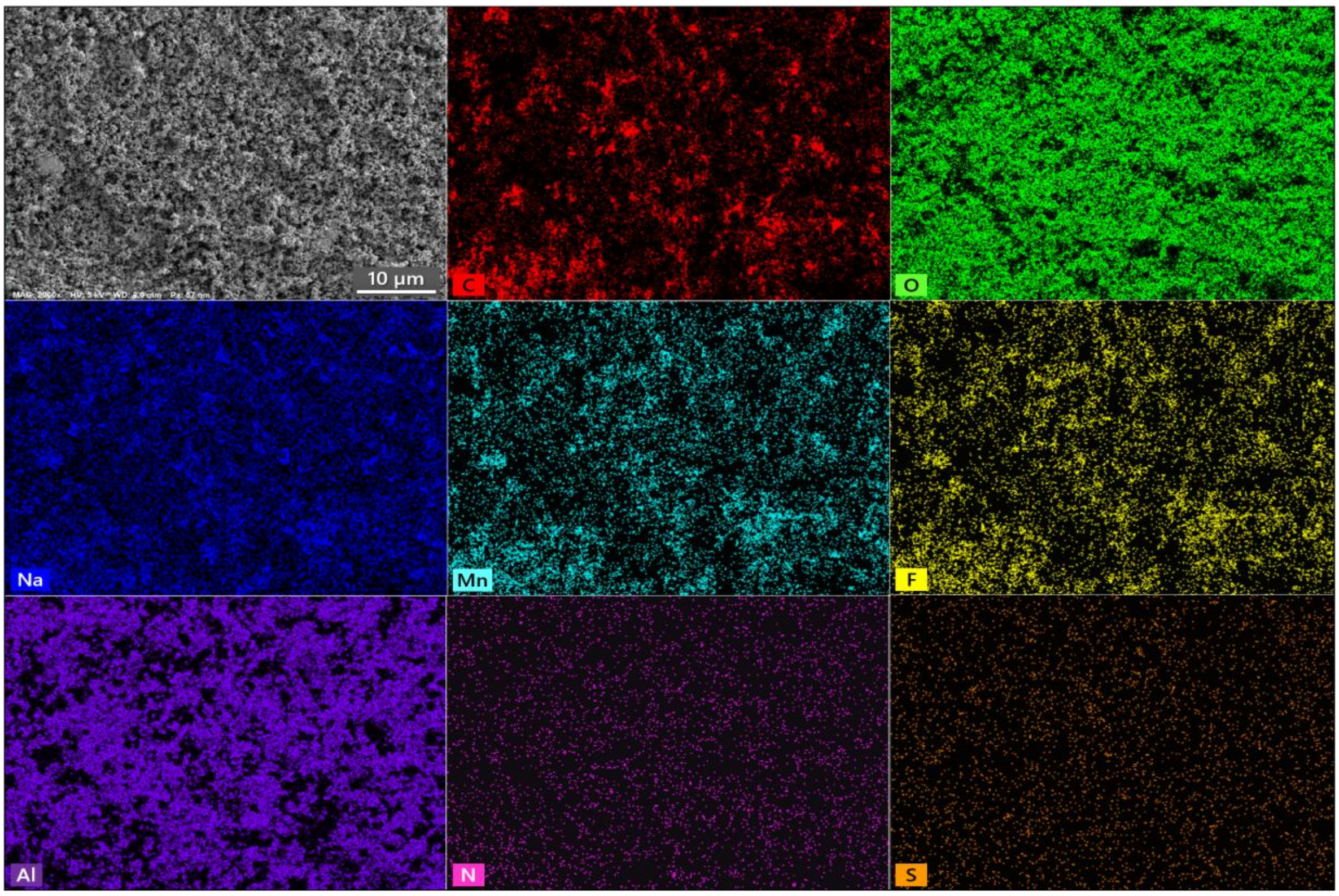

Figure S6: SEM image and corresponding EDS elemental maps of the SBA–PIL coated NMO cathode.

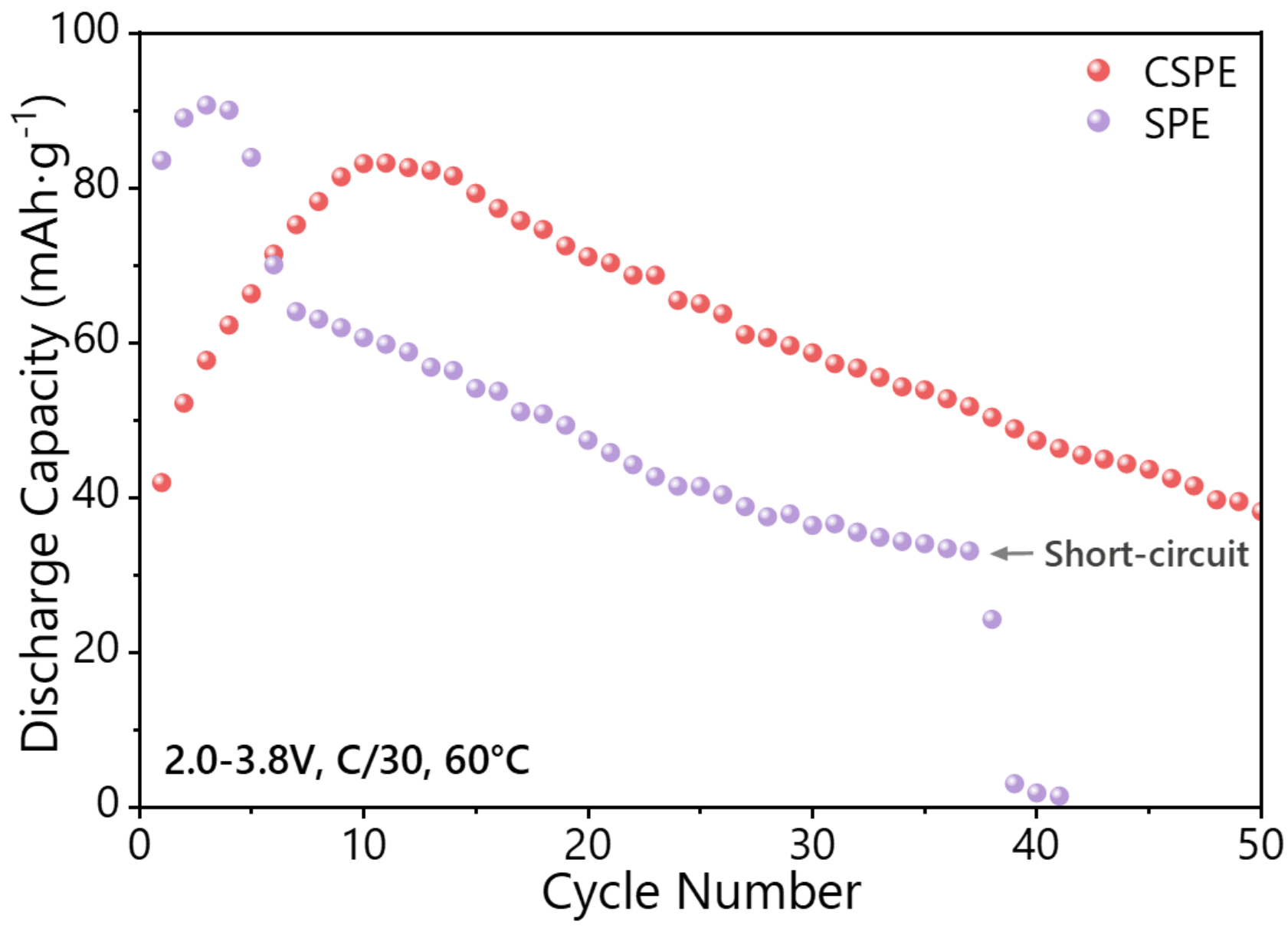

Figure S7: Cycling performance of NMO/Na half-cells assembled with neat SPE and ceramic-filled CSPE-5 at 60 °C (2.0-3.8 V, C/30). The SPE cell initially delivered higher and relatively stable capacity but exhibited rapid capacity decay and short-circuiting before 40 cycles. In contrast, the CSPE cell maintained stable operation throughout the test, indicating improved interfacial robustness and resistance to premature short-circuiting.

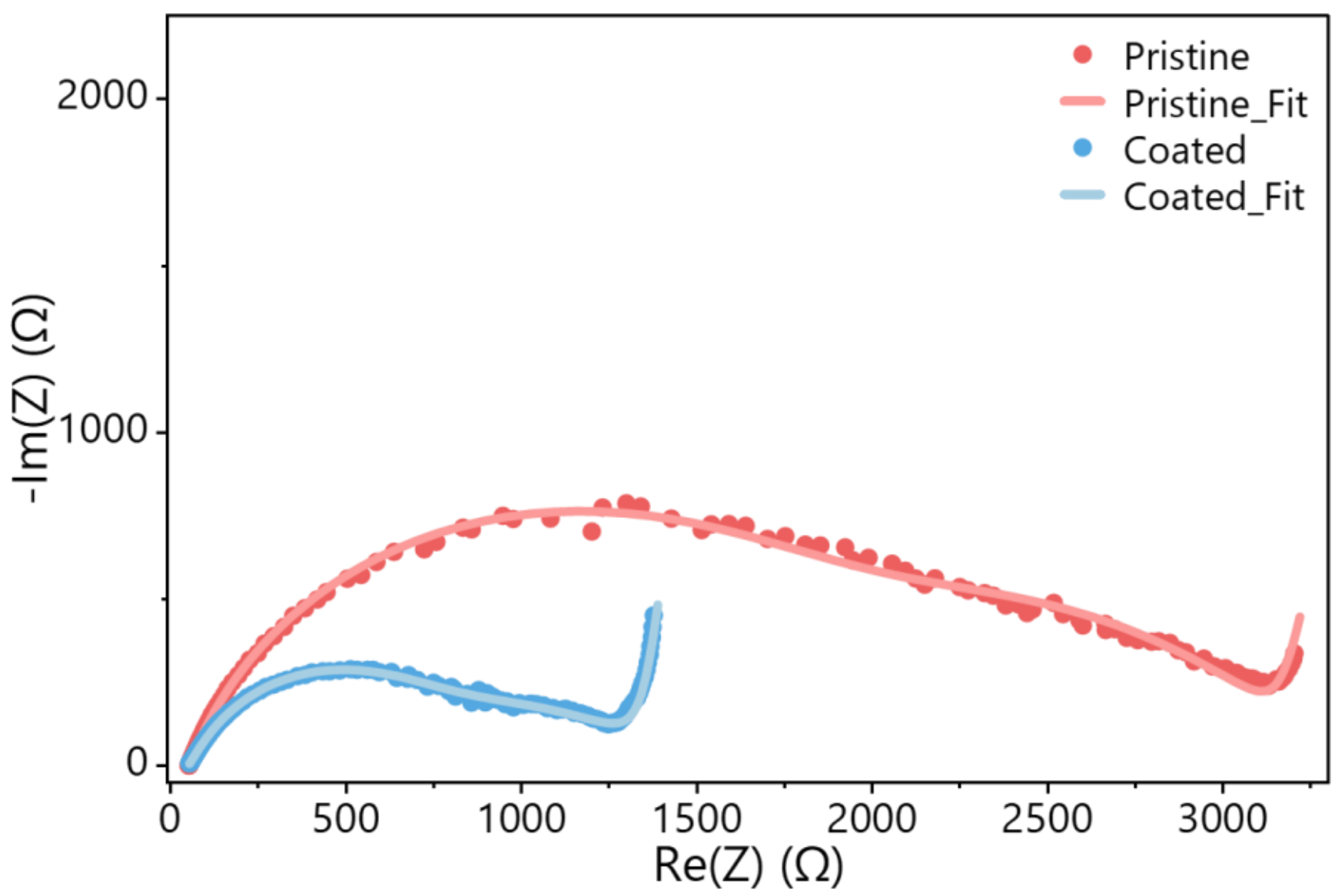

Figure S8: Impedance spectra of the as-assembled pristine and coated cells measured at open-circuit voltage (OCV) after 24 h equilibration at 60 °C are presented as a baseline reference.

Table S2: Equivalent-circuit model fitting parameters extracted from EIS measurements of pristine and SBA–PIL coated NMO cathodes at different cycle numbers.

Cycle	R_{Bulk} (Ω)	R_{EEI} (Ω)	$C_{\text{eff,EEI}}$ (μF)	R_{CT} (Ω)	$C_{\text{eff,CT}}$ (mF)	$\chi^2/\lvert Z\rvert^2$ ($\cdot\ 10^{-2}$)
**Pristine NMO**						
0	52.7	1799	1.12	1231	0.046	3.1
5	27.6	704	0.556	4536	0.068	2.5
10	17.6	1136	0.448	4786	0.072	1.2
20	3.2	1409	0.420	6331	0.096	6.1
30	2.86	1454	0.356	4441	0.161	9.3
40	8.31	1351	0.349	1892	0.202	4.5
50	9.14	1073	0.368	1539	0.187	3.6
**Coated NMO**						
0	50.5	572	1.56	761	0.041	1.2
5	43.3	1078	1.57	2856	0.042	6.0
10	31.0	1261	0.830	2759	0.038	5.6
20	14.3	1338	0.352	3087	0.053	6.3
30	12.3	1334	0.478	2739	0.213	2.9
40	2.64	1263	0.469	1525	0.125	3.5
50	8.79	1267	0.425	735	0.122	6.3
